# Supplementary material for: Spatio-temporal analysis of human brucellosis distribution in Neyshabur, Iran from 2015 to 2022, a cross-sectional study
Source: PLoS One. 2025 Feb 18;20(2):e0318503. doi: 10.1371/journal.pone.0318503 (PMC11835317; doi:10.1371/journal.pone.0318503)
Supplement: Attachment — Submitted filename: PONE-D-24-16267_reviewer.pdf [file pone.0318503.s002.pdf]

PLOS ONE

Spatio-temporal analysis of human brucellosis distribution in Neyshabur, Iran from 2015 to 2022

--Manuscript Draft--

**Manuscript Number:**	PONE-D-24-16267
**Article Type:**	Research Article
**Full Title:**	Spatio-temporal analysis of human brucellosis distribution in Neyshabur, Iran from 2015 to 2022
**Short Title:**	Spatio-temporal analysis of human brucellosis distribution in Neyshabur, Iran from 2015 to 2022
**Corresponding Author:**	Mohammad Maroosi, ph.D<br>Neyshabur University of Medical Sciences<br>Neyshabur, Khorasan Razavi IRAN, ISLAMIC REPUBLIC OF
**Keywords:**	Human brucellosis<br>Spatio-Temporal Analysis<br>Spatial Analysis<br>Cluster Analysis<br>Geographic Information Systems
**Abstract:**	Background: Human brucellosis is a global endemic disease and a significant public health concern. This cross-sectional study aims to explore the characteristics of human brucellosis, as well as its spatial and temporal clustering.<br>Methods: The data on human brucellosis was collected from the Center for Prevention and Control of Contagious Diseases, under the supervision of the Vice-Chancellor for Health of Neyshabur University of Medical Sciences from 2015 to 2022. Spatial autocorrelation analysis using Arc GIS Pro 3.0.1 and statistical analysis employed to identify trends in changes in the spatial and temporal distribution and human characteristics of brucellosis.<br>Results : During the study period, a total of 4568 brucellosis patients were reported. The incidence of disease was higher in men with 55.37%. The majority of patients belonged to the 30 to 39-year-old age group. Temporal clustering analysis revealed the peak incidence between March and June. Spatial clustering analysis indicated a higher prevalence in the southern and southeast districts of Neyshabur. The results of Global Moran’s I analysis indicated that the distribution of brucellosis was not clustered in Neyshabur.<br>Conclusion:<br>It is crucial to understand the seasonal trends in brucellosis to implement effective prevention strategies. By analyzing the data, our study shows a high infection prevalence from March to July between 2015 and 2022 due to contact with infected livestock. Given the increased prevalence, the emphasis is on preventive strategies including livestock vaccination, and educating families and farmers are needed to mitigate the risk.<br>Key words: Human brucellosis, Spatio-Temporal Analysis, Spatial Analysis, Cluster Analysis, Geographic Information Systems
**Order of Authors:**	Mohammad Maroosi, ph.D<br>Ayoub FathAbadi<br>Fatemeh Khorashadizadeh<br>Reza Darrudi
**Additional Information:**	
**Question**	**Response**
**Financial Disclosure**<br><br>Enter a financial disclosure statement that describes the sources of funding for the	Yes

work included in this submission. Review the submission guidelines for detailed requirements. View published research articles from *PLOS ONE* for specific examples.<br><br>This statement is required for submission and **will appear in the published article** if the submission is accepted. Please make sure it is accurate.<br><br>**Funded studies**<br>Enter a statement with the following details:<br>• Initials of the authors who received each award<br>• Grant numbers awarded to each author<br>• The full name of each funder<br>• URL of each funder website<br>• Did the sponsors or funders play any role in the study design, data collection and analysis, decision to publish, or preparation of the manuscript?<br><br>Did you receive funding for this work?	
Please add funding details.<br>as follow-up to "**Financial Disclosure**<br><br>Enter a financial disclosure statement that describes the sources of funding for the work included in this submission. Review the submission guidelines for detailed requirements. View published research articles from *PLOS ONE* for specific examples.<br><br>This statement is required for submission and **will appear in the published article** if the submission is accepted. Please make sure it is accurate.	This study was supported by the Student Research Committee (SRC) funds of Neyshabur University of Medical Sciences (NUMS).

**Funded studies**<br>Enter a statement with the following details:<br>• Initials of the authors who received each award<br>• Grant numbers awarded to each author<br>• The full name of each funder<br>• URL of each funder website<br>• Did the sponsors or funders play any role in the study design, data collection and analysis, decision to publish, or preparation of the manuscript?<br><br>Did you receive funding for this work?"	
Please select the country of your main research funder (please select carefully as in some cases this is used in fee calculation).<br>as follow-up to "**Financial Disclosure**<br><br>Enter a financial disclosure statement that describes the sources of funding for the work included in this submission. Review the submission guidelines for detailed requirements. View published research articles from *PLOS ONE* for specific examples.<br><br>This statement is required for submission and **will appear in the published article** if the submission is accepted. Please make sure it is accurate.<br><br>**Funded studies**<br>Enter a statement with the following details:<br>• Initials of the authors who received each award<br>• Grant numbers awarded to each author<br>• The full name of each funder<br>• URL of each funder website<br>• Did the sponsors or funders play any role in the study design, data collection and analysis, decision to publish, or preparation of the manuscript?<br><br>Did you receive funding for this work?"	IRAN, ISLAMIC REPUBLIC OF - IR
**Competing Interests**	The authors have declared that no competing interests exist.

Use the instructions below to enter a competing interest statement for this submission. On behalf of all authors, disclose any competing interests that could be perceived to bias this work—acknowledging all financial support and any other relevant financial or non-financial competing interests.<br><br>This statement is required for submission and **will appear in the published article** if the submission is accepted. Please make sure it is accurate and that any funding sources listed in your Funding Information later in the submission form are also declared in your Financial Disclosure statement.<br><br>View published research articles from *PLOS ONE* for specific examples.<br><br>**NO authors have competing interests**<br><br>Enter: *The authors have declared that no competing interests exist.*<br><br>**Authors with competing interests**<br><br>Enter competing interest details beginning with this statement:<br><br>*I have read the journal's policy and the authors of this manuscript have the following competing interests: [insert competing interests here]*<br><br>* typeset	
**Ethics Statement**<br><br>Enter an ethics statement for this submission. This statement is required if the study involved:<br><br>• Human participants<br>• Human specimens or tissue<br>• Vertebrate animals or cephalopods<br>• Vertebrate embryos or tissues	The present study, approved by the Research Ethical Committee of Neyshabur University of Medical Sciences under the ethical code IR.NUMS.REC.1401.042, is in compliance with ethical standards.

• Field research<br><br>Write "N/A" if the submission does not require an ethics statement.<br><br>General guidance is provided below. Consult the submission guidelines for detailed instructions. **Make sure that all information entered here is included in the Methods section of the manuscript.**	

Powered by Editorial Manager® and ProduXion Manager® from Aries Systems Corporation

**Format for specific study types**<br><br>**Human Subject Research (involving human participants and/or tissue)**<br>• Give the name of the institutional review board or ethics committee that approved the study<br>• Include the approval number and/or a statement indicating approval of this research<br>• Indicate the form of consent obtained (written/oral) or the reason that consent was not obtained (e.g. the data were analyzed anonymously)<br><br>**Animal Research (involving vertebrate animals, embryos or tissues)**<br>• Provide the name of the Institutional Animal Care and Use Committee (IACUC) or other relevant ethics board that reviewed the study protocol, and indicate whether they approved this research or granted a formal waiver of ethical approval<br>• Include an approval number if one was obtained<br>• If the study involved *non-human primates*, add *additional details* about animal welfare and steps taken to ameliorate suffering<br>• If anesthesia, euthanasia, or any kind of animal sacrifice is part of the study, include briefly which substances and/or methods were applied<br><br>**Field Research**<br><br>Include the following details if this study involves the collection of plant, animal, or other materials from a natural setting:<br>• Field permit number<br>• Name of the institution or relevant body that granted permission	
**Data Availability**<br><br>Authors are required to make all data underlying the findings described fully available, without restriction, and from the time of publication. PLOS allows rare exceptions to address legal and ethical concerns. See the PLOS Data Policy and FAQ for detailed information.	No - some restrictions will apply

A Data Availability Statement describing where the data can be found is required at submission. Your answers to this question constitute the Data Availability Statement and **will be published in the article**, if accepted.<br><br>**Important:** Stating 'data available on request from the author' is not sufficient. If your data are only available upon request, select 'No' for the first question and explain your exceptional situation in the text box.<br><br>Do the authors confirm that all data underlying the findings described in their manuscript are fully available without restriction?	
**Describe where the data may be found in full sentences. If you are copying our sample text, replace any instances of XXX with the appropriate details.**<br><br>• If the data are **held or will be held in a public repository**, include URLs, accession numbers or DOIs. If this information will only be available after acceptance, indicate this by ticking the box below. For example: *All XXX files are available from the XXX database (accession number(s) XXX, XXX.).*<br>• If the data are all contained **within the manuscript and/or Supporting Information files**, enter the following: *All relevant data are within the manuscript and its Supporting Information files.*<br>• If neither of these applies but you are able to provide **details of access elsewhere**, with or without limitations, please do so. For example:<br><br>*Data cannot be shared publicly because of [XXX]. Data are available from the XXX Institutional Data Access / Ethics Committee (contact via XXX) for researchers who meet the criteria for access to confidential data.*<br><br>*The data underlying the results presented in the study are available from (include the name of the third party*	The data on human brucellosis cases was obtained from the Center for Prevention and Control of Contagious Diseases, under the supervision of the Vice-Chancellor for Health of Neyshabur University of Medical Sciences. However, full availability of the data needs to be approved

and contact information or URL).<br>• This text is appropriate if the data are owned by a third party and authors do not have permission to share the data.<br><br>* typeset	
Additional data availability information:	

Manuscript Click here to access/download;Manuscript;Manuscript.docx

Spatio-temporal analysis of human brucellosis distribution in Neyshabur, Iran from 2015 to 2022

Ayoub Fathabadi[1¶], Fatemeh Khorashadizadeh[2&], Reza Darrudi[1&], Mohammad Maroosi[3*]

[1] Department of Health Information Technology, Faculty of Health and Paramedicine,

Neyshabur University of Medical Sciences, Neyshabur, Iran

[2] Department of Epidemiology and Biostatistics,

Neyshabur University of Medical Sciences, Neyshabur, Iran

[3] Department of Environmental Health Engineering, Faculty of Health and Paramedicine, Neyshabur University of Medical Sciences, Neyshabur, Iran

* Corresponding author
E-mail: maroosim@nums.ac.ir

Abstract

Background: Human brucellosis is a global endemic disease and a significant public health concern. This cross-sectional study aims to explore the characteristics of human brucellosis, as well as its spatial and temporal clustering.

Methods: The data on human brucellosis was collected from the Center for Prevention and Control of Contagious Diseases, under the supervision of the Vice-Chancellor for Health of Neyshabur University of Medical Sciences from 2015 to 2022. Spatial autocorrelation analysis using Arc GIS Pro 3.0.1 and statistical analysis employed to identify trends in changes in the spatial and temporal distribution and human characteristics of brucellosis.

Results: During the study period, a total of 4568 brucellosis patients were reported. The incidence of disease was higher in men with 55.37%. The majority of patients belonged to the 30 to 39-year-old age group. Temporal clustering analysis revealed the peak incidence between March and June. Spatial clustering analysis indicated a higher prevalence in the southern and southeast districts of Neyshabur. The results of Global Moran's I analysis indicated that the distribution of brucellosis was not clustered in Neyshabur.

Conclusion:

It is crucial to understand the seasonal trends in brucellosis to implement effective prevention strategies. By analyzing the data, our study shows a high infection prevalence from March to July between 2015 and 2022 due to contact with infected livestock. Given the increased prevalence, the emphasis is on preventive strategies including livestock vaccination, and educating families and farmers are needed to mitigate the risk.

Key words: Human brucellosis, Spatio-Temporal Analysis, Spatial Analysis, Cluster Analysis, Geographic Information Systems

Introduction

Human Brucellosis is one of the seven neglected and under-detected acute febrile diseases that remains one of the major causes of morbidity throughout the world[1]. Research has established that brucellosis as a bacterial infection, transmitted to human from ingestion of contaminated dairy or contact with infected animals, is an important risk factor in human health. This relates to occupational, especially working with animals, ethnic and regional aspects of individuals' lifestyle. There is now a strong body of evidence to support that brucellosis can cause a reduction in quality of life[2], herd productivity, and food security[3] Considering human health, this disease affects the body's immune system leading to flu-like symptoms such as fever, weakness, malaise, weight loss and fatigue[4]. The medical, veterinary and socioeconomic implications are emphasized as the other significant public health concerns associated with brucellosis[4]. Brucellosis prevalence has been reported to be highest in some areas such as Central Asia, Mediterranean countries, Latin America, and sub-Saharan Africa. Due to the increasing trends in international traveling, trade and migration, the prevalence of the disease has increased in all ages of the population[5]. The confirmed cases are estimated in studies in various Asian countries with variable prevalence, including India (16.7%), Turkey (8.8%), Saudi Arabia (12.7%), Iraq (12.6%), and Iran (6.59%)[6]. However, a significant reduction in the incidence of cases has been observed in some countries, including Australia, New Zealand, and Japan[3]. This suggests that the prevention or even elimination of human brucellosis may be feasible which requires the prevention programs to achieve the desired outcomes. Iran, also, is endemic for human brucellosis, notably prevalent in areas

where people reside in close contact with infected animals or animal products.[7, 8]. By 2017, the annual disease rate was estimated at one case per 100,000 people that the highest rate was reported in the western and northern regions of the country[9]. With emergence of the new technologies in the field of health, Geographical Information Systems (GIS) have demonstrated the capability to discover the new epidemic pattern and hotspot areas to precisely provide policy guidance for brucellosis control[10] and significant reduction associated with the costs, care and prevention programs based on the geographical data[11]. Using the data from GIS in epidemiological investigations into the prevalence of brucellosis in Iran, Shirzadi et.al (2021) identified the high-priority areas to implement the targeted interventions to control the disease[12]. According to Azizi et.al (2019), because of traditional consumption habits of unpasteurized fresh and local dairy products, Neyshabur stands out as a highly polluted and susceptible region to brucellosis, considering the pattern of livestock distribution, the spatial distribution of this disease varies in villages[13].

In the present study, we employed a spatiotemporal distribution model to analyze local surveillance data on human brucellosis. Moreover, we focus on investigating the human and spatiotemporal distribution characteristics, as well as correlations of human brucellosis epidemics in Neyshabur, the second-largest city of Razavi Khorasan Province in the Northeast of Iran, between 2015 and 2022.

Materials and methods

Study area

This cross-sectional study was conducted in Neyshabur, the north-eastern region of Iran, with a population of 451,780 [14]. Neyshabur with a 5.653 km^2 area, located in 36° 25′ 26″ N, 58° 37′ 31″ E[15], is the second-largest city in the Khorasan Razavi

province and the third-largest city in Eastern Iran. Neyshabur has 418 habitable villages organized into census blocks, which are the smallest spatial units in the cities of Iran. For this study, rural districts, a political division in Iran, were utilized as the geographical scale for conducting the spatial analyses.

Data sources and patients

The data was collected from two sources comprising both non-spatial and spatial. Firstly, the data on human brucellosis cases was obtained from the Center for Prevention and Control of Contagious Diseases, under the supervision of the Vice-Chancellor for Health of Neyshabur University of Medical Sciences from 2015 to 2022. Secondly, the spatial divisions map of Neyshabur area was acquired through the municipal authorities. Because the lack of registered latitude and longitude for human brucellosis cases, we had to geocode patients' addresses using Google My Maps. To ensure patient privacy, we used the geomasked technique. Each patient's address was randomly assigned within a radius of half a kilometer around his/her home. Because of the study design that was executed at the district level, the probability pointing address of patients did not limited our study.

Ethics approval and consent to participate

This retrospective study, approved by the Research Ethical Committee of Neyshabur University of Medical Sciences under the ethical code IR.NUMS.REC.1401.042, is in compliance with ethical standards. After the conformation of the study, the data related to human participants received on April 9, 2023 was received by the study team from the Center for Prevention and Control of Contagious Diseases in a as blinded format. This means that data were de-identified at the source by trained personnel. Additionally, the study

team is committed to using these data only for the present study, and no one outside the study team has access to the data.

Anselin Local Moran's I

Anselin's Local Moran I, a local spatial autocorrelation derived from the Moran's I statistic, was applied in this study to identify significant spatial clusters and outliers of districts exhibiting high or low incidences of human brucellosis. This method calculates the Moran's I value by creating a neighborhood around each district and compares it to the overall study area. When less than 5% of the Local Moran's I values, generated from permutations, display more clustering than the original data, then the significance is determined[16]. Moreover, whereas low values in clusters indicate cold spots, hot spots refer to high values.

Global Moran's I

Global Moran's I is a spatial analysis tool used to simultaneously measure spatial autocorrelation in features[16] .It's employed to assess the distribution pattern of human brucellosis cases in the study area to identify whether the distribution shows clustering, dispersion, or randomness. The null hypothesis states that the spatial distribution of human brucellosis cases is random, and this hypothesis can be rejected if the Z-score exceeds 1.96 and P-value is less than 0.05, indicating statistical significance and the presence of spatial autocorrelation [17]. Furthermore, while Moran's I value near -1.0 indicates a dissimilar cluster (dispersion), an index value near +1.0 suggests a similar cluster (clustering) of human brucellosis distribution at the level of rural districts. Otherwise, a randomized distribution is inferred.

Results

A total of 4568 cases of human brucellosis were registered in Neyshabur from 2015 to 2022. As shown in Fig 1, the annual frequency and trend of the disease reveals an ascending from 2018 to 2022 while the other years have no constant trends. Our results show that the prevalence was the highest in summer with 1588 patients (34.76%) and the lowest in the winter with 583 patients (12.76%). Moreover, the prevalence in the spring and autumn was accounted for 1546 (33.85%) and 851 (18.63%) respectively. Similarly, Fig 2 illustrates the seasonal trend for this disease showing an increase from December, peaking up from March to June then a declining trend in a number of patients of the total patients, 88.9 % lived in rural and 11.1 % lived in urban areas. Men constituted 55.37% of the cases and women were 44.63%. The overall median age was 38 years (interquartile range (IQR)= 25-52). There was a significant relationship between patients' age and gender ($P<0.001$). Fig 3 shows distribution of patient's age. This implies that the disease affects all age groups and is not specific to any particular age group. The majority of patients fell into the 30 to 39-year-old age group. Based on the self-reported information provided by patients, 86.01% had direct interaction with livestock at their home or work, 67.59% reported consuming raw dairy products, 50.75% were informed about the vaccination status of livestock for brucellosis, particularly those with which they had interaction or from which they had consume raw dairy products. Additionally, the study showed that the prevalence of brucellosis among the household members was17.37%.

Fig 4 demonstrates the geographical distribution of human brucellosis. It shows a higher prevalence in the southern and southeast districts of Neyshabur. In Fig 5, the spatial statistics of Anselin's Local Moran I (cluster and outlier analysis) for human brucellosis at the district level of Neyshabur are presented. The analysis reveals that, in certain years, the northern districts exhibited significantly low-low clusters (or cold

spots) of human brucellosis in the study area. Furthermore, high-high clusters (or hot spots) were observed in the southern area of the study for two years (2017-2019). Additionally, low high outliers were identified in the southern area of Neyshabur for three years (2017-2019-2021). No significant clusters or outliers were observed in other areas. Table 1 presents the calculated Moran's I value, Z score, and P value for the distribution of human brucellosis in Neyshabur from 2015 to 2022, using district units to conduct the global autocorrelation analysis. The Moran's I value scope were 0.2737 that variable between 0.0123 to 0.2860 in during of study, respectively, the Z score values were less than 1.96 ($P > .05$), indicating that the incidence of human brucellosis in Neyshabur from 2015 to 2022 had a random distribution (not clustered).

Table 1. Global auto-correlation Moran's I value of human brucellosis in Neyshabur city, Iran during 2015 to 2022

Year	Moran's I	Variance	Z score	P value	Aggregation
2015	0.184453	0.048362	1.091377	0.275107	No
2016	0.110425	0.048846	0.751000	0.452653	No
2017	0.012333	0.050060	0.303427	0.761565	No
2018	0.055398	0.047653	0.508272	0.611263	No
2019	0.240794	0.044657	1.402355	0.160809	No
2020	0.245693	0.049658	1.351850	0.176423	No
2021	0.146019	0.048417	0.916091	0.359619	No
2022	0.286051	0.049117	1.541381	0.123224	No

Discussion

The purpose of the present study is to investigate the spatiotemporal distribution of human brucellosis in Neyshabur, using a spatial panel model to analyze the different factors affecting the prevalence of the disease in diverse geographical areas and to provide scientific strategies for the health authorities to control and prevent the disease.

The results of our temporal cluster analysis demonstrated the presence of a distinct seasonality in human brucellosis. There was a seasonal trend in the prevalence of the disease with the highest rate (0.34) in summer and the lowest (0.12) in winter, reaching its peak from May to June that it can be potentially attributed to the breeding period of the animals. This result ties well with previous studies wherein the investigations into human brucellosis prevalence in different geographical areas reported the highest rate in summer[18]. However, the lowest rates were reported to vary, occurring either in autumn or winter[19]. Moreover, several studies have identified temperature as a risk factor for human brucellosis, indicating that the likelihood of incidence rises with increasing temperatures[20-22]. That aligns with our findings; the disease prevalence increases as the warmer seasons of the year (spring and summer) approach and notably decreases during the colder seasons of the year.

Considering age and gender, although the cases were not clustered in a specific age group, we found that a higher prevalence of human brucellosis among individuals in their thirties (20.09%) compared to other age brackets, with men being the most affected. This is similar to the findings of a study which were conducted to analyze the age effect on the prevalence rates of brucellosis in different geographical areas[23]. Compared to our findings, a seroepidemiological study on 323 participants working in butcheries, slaughter rooms, and breeding farms observed the lowest rate of infection (6.3%) in 30-39 years[24]. Moreover, for gender, the majority of previous studies detected a higher positivity rate in women than males[25, 26]. The difference in disease prevalence between both genders may be associated with their engagement in working with livestock and their products. However, whether the risk for infection sexually to brucellosis is higher for males or females has not been established yet. Regarding manual milking, using its raw products, and keeping animals near the

homes in most rural areas, our findings showed a rate of 88% of the human brucellosis cases. In contrast, the previous reports showed that the prevalence of the disease was 82.2% in Iran[7], 63.5% in Saudi Arabia[27], and 88.18% in China[28]. Based on these findings, we hypothesize that the rural lifestyle in Iran, characterized by the use of traditional animal husbandry and agricultural practices for the sustenance of the majority of villagers, exposes them to an increased risk.

The results of the spatial clustering analysis of human brucellosis incidence in nineteen districts across Neyshabur, Iran, revealed the existence of a spatial cluster in each year (excluding 2016) from 2015 to 2022. The distribution of clustered districts was similar throughout the study years. The findings revealed the existence of clusters in both southern and northern districts. The spatial clusters seem to be contingent on the condition of neighboring districts, while no spatial clusters were detected in the central districts. This may indicate that central districts did not have a similar incidence of human brucellosis cases. In the northern region, especially in the northwest, low-low clusters are observable almost every year. It seems that human brucellosis is non-endemic and does not pose a significant threat to the human population in these districts. The high incidence of human brucellosis cases was observed in southern regions, particularly in southwest districts. A High-High cluster

was identified in 2017 and 2019. The analysis indicates that these districts harbor the potential for an outbreak of human brucellosis, posing a risk of significant and irreversible health and economic repercussions for the local population. The results of the Spatial Autocorrelation analysis (Global Moran's I) of human brucellosis in Neyshabur from 2015 to 2022 suggest that the distribution of human brucellosis cases exhibits randomness and does not demonstrate a discernible spatial pattern. In reality, the incidence trend of human brucellosis does not adhere to a discernible pattern, thereby presenting challenges in forecasting future conditions.

Conclusions

For the implementation of well prevention strategies Information about trends of incidence can be critical to management of resources in specific space and time. The results of our spatiotemporal cluster analysis demonstrate the presence of a distinct seasonality in human brucellosis. The period of primary prevalence extended from March to July, from 2015 to 2022 in Neyshabur, Iran. Given the heightened risk of contracting the disease through contact with blood, placenta, fetuses, and uterine secretions, it seems that these months present higher risk factors for human infection. Considering that the primary and most effective method to prevent Human Brucellosis is to interrupt the transmission cycle from infected livestock to humans, the rate is much lower than anticipated. In addition, a majority of the patients have familial ties to animal husbandry as a traditional occupation in rural areas. Consequently, it is anticipated that in the forthcoming years, greater emphasis will be placed on livestock vaccination in rural areas with high infection rates. The consumption of raw dairy products among patients suggests a deficiency in education and awareness concerning diseases such as human brucellosis, particularly among women, children, and the elderly, who have limited direct contact with infected livestock. Therefore, there is a necessity for educational initiatives aimed at preventing the contraction of human brucellosis, with a specific focus on mothers, who hold a pivotal role in the family's nutritional well-being. It seems that optimizing livestock vaccination can reduce the likelihood of contracting brucellosis as an occupational hazard for livestock farmers while instructing the target groups to refrain from using unpasteurized dairy products can alleviate the risk of brucellosis transmission within families.

Limitations & Strength

Due to inadequate accurate registration of the livestock numbers by districts, the lack of access to local animal vaccination data based on the geographical distribution of livestock husbandry, and the deficiency in measuring the annual rainfall at the district level in Neyshabur, the present study didn't examine the effect of these factors on the annual incidence rate of human brucellosis. Despite these limitations, we indicated the spatial pattern of human brucellosis incidence in Neyshabur and increased our knowledge about the most common causes of the disease in the human population. This information can be used as a scientific resource for revising prevalence strategies to control or eradicate human brucellosis in Neyshabur.

Acknowledgments

We would like to extend our sincere gratitude to Dr. Benyamin Hosseini for his valuable comments and suggestions that allowed us to improve the quality of the study. Additionally, we are especially grateful for the help of Dr. Ensiyeh Mollanoroozy for her expert editing support, who spent hours to strengthen the overall quality of the manuscript beyond correcting grammatical and spelling mistakes.

Funding

This study was supported by the Student Research Committee (SRC) funds of Neyshabur University of Medical Sciences (NUMS).

References

1. Nicoletti P. Brucellosis: past, present and future. Prilozi. 2010;31(1):21-32.
2. Havas KA, Ramishvili M, Navdarashvili A, Hill AE, Tsanava S, Imnadze P, Salman MD. Risk factors associated with human brucellosis in the country of Georgia: a case-control study. Epidemiol Infect. 2013;141(1):45-53.
3. Franc KA, Krecek RC, Häsler BN, Arenas-Gamboa AM. Brucellosis remains a neglected disease in the developing world: a call for interdisciplinary action. BMC Public Health. 2018;18(1):125.
4. Babaie E, Alesheikh AA, Tabasi M. Spatial prediction of human brucellosis (HB) using a GIS-based adaptive neuro-fuzzy inference system (ANFIS). Acta Trop. 2021;220:105951.
5. Bosilkovski M, Edwards MS, Calderwood S. Brucellosis: epidemiology, microbiology, clinical manifestations, and diagnosis. Uptodate, United States. 2019.
6. Khalid HM. Seroprevalence and Associated Risk Factors of Brucellosis Among Human Population in Duhok City, Iraq. Infect Drug Resist. 2023;16:2805-11.
7. Zeinali M, Doosti S, Amiri B, Gouya MM, Godwin GN. Trends in the Epidemiology of Brucellosis Cases in Iran during the Last Decade. Iran J Public Health. 2022;51(12):2791-8.
8. Mostafavi E, Asmand M. Trend of brucellosis in Iran from 1991 to 2008. Iranian Journal of Epidemiology. 2012;8(1).
9. Mirnejad R, Jazi FM, Mostafaei S, Sedighi M. Epidemiology of brucellosis in Iran: A comprehensive systematic review and meta-analysis study. Microb Pathog. 2017;109:239-47.
10. Tao Z, Chen Q, Chen Y, Li Y, Mu D, Yang H, Yin W. Epidemiological Characteristics of Human Brucellosis - China, 2016-2019. China CDC Wkly. 2021;3(6):114-9.
11. Halimi L, Bagheri N, Hoseini B, Hashtarkhani S, Goshayeshi L, Kiani B. Spatial analysis of colorectal cancer incidence in Hamadan Province, Iran: a retrospective cross-sectional study. Applied Spatial Analysis and Policy. 2020;13:293-303.
12. Shirzadi MR, Mohammadi P, Moradi G, Goodarzi E, Khazaei S, Moayed L, Khazaei Z. The Incidence and Geographical Distribution of Brucellosis in Iran Using Geographic Information System and Prediction of its Incidence in 2021. J Prev Med Hyg. 2021;62(3):E635-e4.
13. Azizi Mayvan F, Hashtarkhani S, Latifi M, Darrudi R, Hoseini B. Geographic disparities of brucellosis distribution in Neyshabur between 2014 and 2016: a cross-sectional study based on geographical information systems. J Neyshabur Univ Med Sci. 2019;7(4):53-64.
14. Census of the Islamic Republic of Iran, 1395 (2016)". AMAR (in Persian). The Statistical Center of Iran 2016 [Available from: https://web.archive.org/web/20220402220537/https://www.amar.org.ir/.
15. contributors O. Nishapur County 2023 [Available from: https://geohack.toolforge.org/geohack.php?pagename=Nishapur_County&params=36_25_26_N_58_37_31_E_dim:60km_type:city(451780)_region:IR-09.
16. Anselin L. Local indicators of spatial association—LISA. Geographical analysis. 1995;27(2):93-115.
17. Amsalu ET, Akalu TY, Gelaye KA. Spatial distribution and determinants of acute respiratory infection among under-five children in Ethiopia: Ethiopian Demographic Health Survey 2016. PloS one. 2019;14(4):e0215572.
18. Sun ZX, Wang Y, Li YJ, Yu SH, Wu W, Huang DS, Guan P. Socioeconomic, meteorological factors and spatiotemporal distribution of human brucellosis in China between 2004 and 2019-A study based on spatial panel model. PLoS Negl Trop Dis. 2023;17(11):e0011765.
19. Moosazadeh M, Abedi G, Kheradmand M, Safiri S, Nikaeen R. Seasonal pattern of brucellosis in Iran: A systematic review and meta-analysis. 2016.
20. Gebreyes WA, Dupouy-Camet J, Newport MJ, Oliveira CJ, Schlesinger LS, Saif YM, et al. The global one health paradigm: challenges and opportunities for tackling infectious diseases at the human, animal, and environment interface in low-resource settings. PLoS Negl Trop Dis. 2014;8(11):e3257.
21. Godfroid J. Brucellosis in livestock and wildlife: zoonotic diseases without pandemic potential in need of innovative one health approaches. Arch Public Health. 2017;75:34.

22. Hayoun MA ME, Shorman M. Brucellosis 2023 [Available from: Hayoun MA, Muco E, Shorman M. Brucellosis. [Updated 2023 Apr 29]. In: StatPearls [Internet]. Treasure Island (FL): StatPearls Publishing; 2023 Jan-. Available from: https://www.ncbi.nlm.nih.gov/books/NBK441831/.
23. Abdelkader A, Reda BA, Karima G. Prevalence of human brucellosis in the southern Zone of Sidi-Bel-Abbes, Algeria. Archives of Clinical and Medical Case Reports. 2018;2(2):56-64.
24. Mufinda FC, Boinas F, Nunes C. Prevalence and factors associated with human brucellosis in livestock professionals. Rev Saude Publica. 2017;51:57.
25. Alqaseer K, Al-Khafajy AAM, Almkhadhree EAK. Serological and Molecular Detection of Human Brucellosis in Rural Areas in Wasit Province, Iraq. Arch Razi Inst. 2023;78(1):369-78.
26. Hassan L, Ali S, Syed MA, Shah AA, Abbasi SA, Tabassum S, et al. Risk Factors for Acute Brucellosis in Patients on the Day of Admission at Selected Hospitals of Abbottabad, Pakistan. Front Public Health. 2021;9:669278.
27. Elbeltagy KE. An epidemiological profile of brucellosis in Tabuk Province, Saudi Arabia. East Mediterr Health J. 2001;7(4-5):791-8.
28. Liu B, Liu G, Ma X, Wang F, Zhang R, Zhou P, et al. Epidemiology, clinical manifestations, and laboratory findings of 1,590 human brucellosis cases in Ningxia, China. Front Microbiol. 2023;14:1259479.
