# Supplementary material for: Spatio-temporal analysis of human brucellosis distribution in Neyshabur, Iran from 2015 to 2022, a cross-sectional study
Source: PLoS One. 2025 Feb 18;20(2):e0318503. doi: 10.1371/journal.pone.0318503 (PMC11835317; doi:10.1371/journal.pone.0318503)
Supplement: Attachment — Submitted filename: PONE-D-24-16267_R1.pdf [file pone.0318503.s004.pdf]

PLOS ONE

Spatio-temporal analysis of human brucellosis distribution in Neyshabur, Iran from 2015 to 2022, a cross-sectional study

--Manuscript Draft--

**Manuscript Number:**	PONE-D-24-16267R1
**Article Type:**	Research Article
**Full Title:**	Spatio-temporal analysis of human brucellosis distribution in Neyshabur, Iran from 2015 to 2022, a cross-sectional study
**Short Title:**	Spatio-temporal analysis of human brucellosis in Neyshabur, Iran from 2015 to 2022
**Corresponding Author:**	Mohammad Maroosi, ph.D<br>Neyshabur University of Medical Sciences<br>Neyshabur, Khorasan Razavi IRAN, ISLAMIC REPUBLIC OF
**Keywords:**	Human brucellosis<br>Spatio-Temporal Analysis<br>Space-Time Clustering<br>Geographic Information Systems
**Abstract:**	Background: Human brucellosis is a global endemic disease and a significant public health concern. This cross-sectional study aims to explore the characteristics of human brucellosis, as well as its spatial and temporal clustering.<br>Methods: This cross-sectional study with ethical code IR.NUMS.REC.1401.042, analyzed the population infected with human brucellosis in Neyshabur, a city in the east of Iran from 2015 to 2022. Patient data were obtained from the Center for Prevention and Control of Contagious Diseases. Spatial analysis was conducted using Anselin Local Moran's I and Global Moran's I with ArcGIS 10.8.2. Descriptive statistical analyses were performed using SPSS 26.<br>Results: During the study period, a total of 4568 brucellosis patients were reported. The incidence of disease was higher in men with 55.37%. The majority of patients belonged to the 30 to 39-year-old age group. Temporal clustering analysis revealed the peak incidence between March and June. Spatial clustering analysis indicated a higher prevalence in the southern and southeast districts of Neyshabur. The results of Global Moran's I analysis indicated that the distribution of brucellosis was not clustered in Neyshabur.<br>Conclusion:<br>The findings of this study highlight the importance of educational programs for mothers and animal vaccination strategies, particularly in the months leading up to the peak brucellosis season in high-incidence areas. Further research is needed to assess the coverage of livestock vaccination and its impact on human brucellosis rates
**Order of Authors:**	Ayoub FathAbadi
	Fatemeh Khorashadizadeh
	Reza Darrudi
	Mohammad Maroosi, ph.D
**Response to Reviewers:**	Dear Editor,<br><br>Thank you for your feedback on our submission. We understand the importance of data sharing and transparency in research. However, as we previously mentioned, the data used in our study was obtained from the Neyshabur Health Center in the form of an offline document or Excel file. Unfortunately, we are not the owners of this data and cannot provide a direct link for access.<br><br>In addition, the Neyshabur Health Center is a local service-oriented institution without the infrastructure for handling international correspondence, especially in languages other than Persian. There is no established protocol or mechanism in place for responding to data requests in foreign languages or from international researchers. This limitation makes it infeasible to provide any direct contact information for the Health Center regarding data access.

<table>
<tr><td></td><td>That said, while we are unable to make the data publicly available, we are willing to share the data with individual researchers upon request, provided that the request aligns with the ethical guidelines and constraints under which the data was collected. furthermore, the cleaned dataset contain geographial coordinate of patients and it can be considered as sensitive data and restrict data sharing

Given these constraints, we respectfully request that the data sharing requirements be reconsidered in the context of the limitations we face. We assure you that all data used in this study was handled in accordance with local ethical guidelines, and any data sharing beyond our current capabilities is unfortunately not possible.

We appreciate your understanding and look forward to your guidance on how to proceed under these circumstances.

Best regards,
Mohammad Maroosi</td></tr>
<tr><td>Additional Information:</td><td></td></tr>
<tr><td>Question</td><td>Response</td></tr>
<tr><td>Financial Disclosure

Enter a financial disclosure statement that describes the sources of funding for the work included in this submission. Review the submission guidelines for detailed requirements. View published research articles from PLOS ONE for specific examples.

This statement is required for submission and will appear in the published article if the submission is accepted. Please make sure it is accurate.

Funded studies
Enter a statement with the following details:
• Initials of the authors who received each award
• Grant numbers awarded to each author
• The full name of each funder
• URL of each funder website
• Did the sponsors or funders play any role in the study design, data collection and analysis, decision to publish, or preparation of the manuscript?

Did you receive funding for this work?</td><td>Yes</td></tr>
<tr><td>Please add funding details.
as follow-up to "Financial Disclosure

Enter a financial disclosure statement that</td><td>This study was supported by the Student Research Committee (SRC) funds of Neyshabur University of Medical Sciences (NUMS). The funders had no role in study design, data collection and analysis, decision to publish, or preparation of the manuscript.</td></tr>
</table>

describes the sources of funding for the work included in this submission. Review the submission guidelines for detailed requirements. View published research articles from *PLOS ONE* for specific examples.<br><br>This statement is required for submission and **will appear in the published article** if the submission is accepted. Please make sure it is accurate.<br><br>**Funded studies**<br>Enter a statement with the following details:<br>• Initials of the authors who received each award<br>• Grant numbers awarded to each author<br>• The full name of each funder<br>• URL of each funder website<br>• Did the sponsors or funders play any role in the study design, data collection and analysis, decision to publish, or preparation of the manuscript?<br><br>Did you receive funding for this work?"	
Please select the country of your main research funder (please select carefully as in some cases this is used in fee calculation).<br>as follow-up to "**Financial Disclosure**<br><br>Enter a financial disclosure statement that describes the sources of funding for the work included in this submission. Review the submission guidelines for detailed requirements. View published research articles from *PLOS ONE* for specific examples.<br><br>This statement is required for submission and **will appear in the published article** if the submission is accepted. Please make sure it is accurate.	IRAN, ISLAMIC REPUBLIC OF - IR

**Funded studies**<br>Enter a statement with the following details:<br>• Initials of the authors who received each award<br>• Grant numbers awarded to each author<br>• The full name of each funder<br>• URL of each funder website<br>• Did the sponsors or funders play any role in the study design, data collection and analysis, decision to publish, or preparation of the manuscript?<br><br>Did you receive funding for this work?"	
**Competing Interests**<br><br>Use the instructions below to enter a competing interest statement for this submission. On behalf of all authors, disclose any competing interests that could be perceived to bias this work—acknowledging all financial support and any other relevant financial or non-financial competing interests.<br><br>This statement is required for submission and **will appear in the published article** if the submission is accepted. Please make sure it is accurate and that any funding sources listed in your Funding Information later in the submission form are also declared in your Financial Disclosure statement.<br><br>View published research articles from *PLOS ONE* for specific examples.	The authors have declared that no competing interests exist.

**NO authors have competing interests**<br><br>Enter: *The authors have declared that no competing interests exist.*<br><br>**Authors with competing interests**<br><br>Enter competing interest details beginning with this statement:<br><br>*I have read the journal's policy and the authors of this manuscript have the following competing interests: [insert competing interests here]*<br><br>* typeset	
**Ethics Statement**<br><br>Enter an ethics statement for this submission. This statement is required if the study involved:<br><br>• Human participants<br>• Human specimens or tissue<br>• Vertebrate animals or cephalopods<br>• Vertebrate embryos or tissues<br>• Field research<br><br>Write "N/A" if the submission does not require an ethics statement.<br><br>General guidance is provided below. Consult the submission guidelines for detailed instructions. **Make sure that all information entered here is included in the Methods section of the manuscript.**	The present study, approved by the Research Ethical Committee of Neyshabur University of Medical Sciences under the ethical code IR.NUMS.REC.1401.042, is in compliance with ethical standards.

Powered by Editorial Manager® and ProduXion Manager® from Aries Systems Corporation

**Format for specific study types**<br><br>**Human Subject Research (involving human participants and/or tissue)**<br>• Give the name of the institutional review board or ethics committee that approved the study<br>• Include the approval number and/or a statement indicating approval of this research<br>• Indicate the form of consent obtained (written/oral) or the reason that consent was not obtained (e.g. the data were analyzed anonymously)<br><br>**Animal Research (involving vertebrate animals, embryos or tissues)**<br>• Provide the name of the Institutional Animal Care and Use Committee (IACUC) or other relevant ethics board that reviewed the study protocol, and indicate whether they approved this research or granted a formal waiver of ethical approval<br>• Include an approval number if one was obtained<br>• If the study involved *non-human primates*, add *additional details* about animal welfare and steps taken to ameliorate suffering<br>• If anesthesia, euthanasia, or any kind of animal sacrifice is part of the study, include briefly which substances and/or methods were applied<br><br>**Field Research**<br><br>Include the following details if this study involves the collection of plant, animal, or other materials from a natural setting:<br>• Field permit number<br>• Name of the institution or relevant body that granted permission	
**Data Availability**<br><br>Authors are required to make all data underlying the findings described fully available, without restriction, and from the time of publication. PLOS allows rare exceptions to address legal and ethical concerns. See the PLOS Data Policy and FAQ for detailed information.	No - some restrictions will apply

A Data Availability Statement describing where the data can be found is required at submission. Your answers to this question constitute the Data Availability Statement and **will be published in the article**, if accepted.<br><br>**Important:** Stating 'data available on request from the author' is not sufficient. If your data are only available upon request, select 'No' for the first question and explain your exceptional situation in the text box.<br><br>Do the authors confirm that all data underlying the findings described in their manuscript are fully available without restriction?	
**Describe where the data may be found in full sentences. If you are copying our sample text, replace any instances of XXX with the appropriate details.**<br><br>• If the data are **held or will be held in a public repository**, include URLs, accession numbers or DOIs. If this information will only be available after acceptance, indicate this by ticking the box below. For example: *All XXX files are available from the XXX database (accession number(s) XXX, XXX.*).<br>• If the data are all contained **within the manuscript and/or Supporting Information files**, enter the following: *All relevant data are within the manuscript and its Supporting Information files.*<br>• If neither of these applies but you are able to provide **details of access elsewhere**, with or without limitations, please do so. For example:<br><br>*Data cannot be shared publicly because of [XXX]. Data are available from the XXX Institutional Data Access / Ethics Committee (contact via XXX) for researchers who meet the criteria for access to confidential data.*<br><br>*The data underlying the results presented in the study are available from (include the name of the third party*	Data Availability Statement<br><br>The data used in this study was obtained from the Neyshabur Health Center as offline documents and Excel files. As the authors do not own the data, we are unable to provide a direct link or public access. Additionally, the Neyshabur Health Center is a local service-oriented institution that lacks the infrastructure for handling international correspondence, especially in languages other than Persian. There is no established protocol for responding to data requests from international researchers, which limits our ability to facilitate direct contact for data access.<br>The data includes sensitive information such as geographical coordinates of patients, which imposes additional restrictions on sharing due to privacy and ethical considerations. Despite these constraints, the authors are committed to sharing the data with individual researchers upon request, provided that such requests comply with the ethical guidelines and constraints under which the data was collected. The data will be kept safely and securely in a personal and online encrypted cloud repository to ensure persistent or long-term data storage and availability.<br>For inquiries regarding data access, please contact Mohammad Maroosi (maroosim@nums.ac.ir), Ayoub Fathabadi (ayofathabadi@gmail.com), Fatemeh Khorashadizadeh (fkhorashadi89@yahoo.com) and Reza Darrudi (darrudir1@nums.ac.ir). Access requests will be evaluated on a case-by-case basis in accordance with applicable ethical and legal standards, including those outlined in the IRB-approved protocol or patient consent form.<br>Although the authors cannot make their study's data publicly available at the time of publication, all authors commit to make the data underlying the findings described in this study fully available without restriction to those who request the data, in compliance with the PLOS Data Availability policy. For data sets involving personally identifiable information or other sensitive data, data sharing is contingent on the data being handled appropriately by the data requester and in accordance with all applicable local requirements.

and contact information or URL).<br>• This text is appropriate if the data are owned by a third party and authors do not have permission to share the data.<br><br>* typeset	
Additional data availability information:	

Powered by Editorial Manager® and ProduXion Manager® from Aries Systems Corporation

The manuscript Click here to access/download;Manuscript;Manuscript.docx

Spatio-temporal analysis of human brucellosis distribution in Neyshabur, Iran from 2015 to 2022, a cross-sectional study

Ayoub Fathabadi[1¶], Fatemeh Khorashadizadeh[2&], Reza Darrudi[1&], Mohammad Maroosi[3*]

[1] Department of Health Information Technology, Faculty of Health and Paramedicine,

Neyshabur University of Medical Sciences, Neyshabur, Iran

[2] Department of Epidemiology and Biostatistics,

Neyshabur University of Medical Sciences, Neyshabur, Iran

[3] Department of Environmental Health Engineering, Faculty of Health and Paramedicine, Neyshabur University of Medical Sciences, Neyshabur, Iran

* Corresponding author
E-mail: maroosim@nums.ac.ir

Abstract

Background: Human brucellosis is a global endemic disease and a significant public health concern. This cross-sectional study aims to explore the characteristics of human brucellosis, as well as its spatial and temporal clustering.

Methods: This cross-sectional study with ethical code IR.NUMS.REC.1401.042, analyzed the population infected with human brucellosis in Neyshabur, a city in the east of Iran from 2015 to 2022. Patient data were obtained from the Center for Prevention and Control of Contagious Diseases. Spatial analysis was conducted using Anselin Local Moran's I and Global Moran's I with ArcGIS 10.8.2. Descriptive statistical analyses were performed using SPSS 26.

Results: During the study period, a total of 4568 brucellosis patients were reported. The incidence of disease was higher in men with 55.37%. The majority of patients belonged to the 30 to 39-year-old age group. Temporal clustering analysis revealed the peak incidence between March and June. Spatial clustering analysis indicated a higher prevalence in the southern and southeast districts of Neyshabur. The results of Global Moran’s I analysis indicated that the distribution of brucellosis was not clustered in Neyshabur.

Conclusion:

The findings of this study highlight the importance of educational programs for mothers and animal vaccination strategies, particularly in the months leading up to the peak brucellosis season in high-incidence areas. Further

research is needed to assess the coverage of livestock vaccination and its impact on human brucellosis rates

Key words: Human brucellosis, Spatio-Temporal Analysis, Space-Time Clustering, Geographic Information Systems

Introduction

Human Brucellosis is one of the seven neglected and under-detected acute febrile diseases that remains one of the major causes of morbidity throughout the world [1]. It results from the transmission of the brucellosis bacterial agent from infected animals to humans[2]. In populations with the traditional lifestyles, inhalation, direct contact with animal and consumption of unpasteurized dairy products and undercooked meat products are the primary modes transmissions leading to human brucellosis [3, 4]. Human brucellosis is endemic in Iran were many people, especially those in rural areas, commonly keep livestock at their residence [5], This traditional lifestyle significantly contributes to the spread of brucellosis within the Iranian population [6]The incidence of human brucellosis in Iran ranges from 7 to 276 per 100,000 people[7]. Based on previous research, Iran ranks fourth in the world in terms of incidence rate of human brucellosis [8-10]. Although the highest incidences rates of human brucellosis are related to the west and northwest regions of the Iran[7], the most frequent cases of human brucellosis pertain to Razavi Khorasan, the second most populous province in northeast of Iran[11]. According to Norouzinezhad, the

incidence of human brucellosis was more than 20 per 100,000 people in Razavi Khorasan between 2009-2016[12]. This relates to occupational, especially working with animals, ethnic and regional aspects of individuals' lifestyle. There is now a strong body of evidence to support that brucellosis can cause a reduction in quality of life[13], herd productivity, and food security[14] Considering human health, this disease affects the body's immune system leading to flu-like symptoms such as fever, weakness, malaise, weight loss and fatigue[15]. The medical, veterinary and socioeconomic implications are emphasized as the other significant public health concerns associated with brucellosis[15]. Brucellosis prevalence has been reported to be highest in some areas such as Central Asia, Mediterranean countries, Latin America, and sub-Saharan Africa. Due to the increasing trends in international traveling, trade and migration, the prevalence of the disease has increased in all ages of the population[16]. The confirmed cases are estimated in studies in various Asian countries with variable prevalence, including India (16.7%), Turkey (8.8%), Saudi Arabia (12.7%), Iraq (12.6%), and Iran (6.59%)[17]. However, a significant reduction in the incidence of cases has been observed in some countries, including Australia, New Zealand, and Japan[14]. This suggests that the prevention or even elimination of human brucellosis may be feasible which requires the prevention programs to achieve the desired outcomes. Iran, also, is endemic for human brucellosis, notably prevalent in areas where people reside in close contact with infected animals or animal products.[10, 18]. By 2017, the annual disease rate was estimated at one case per 100,000 people that the highest rate was reported in the western and northern regions of the country[7]. According to the Iranian Ministry of Health (MOH), Razavi

Khorasan province, with an average of 21–30 per 100,000 people annually, is among the provinces with the highest incidence rate of human brucellosis in Iran [18]. Neyshabur, the second-largest city in the province, has over350 villages[19], where traditional livestock breeding is common. This increases risk of infection for all members of family due to direct contact with contaminated domestic animals. Further, prevalence of brucellosis varies by time and place[20, 21]. Between 2009 and 2016, the incidence of human brucellosis in Neyshabur ranged from 25.66 to 73.04 cases per 100,000 people [12]. increasing public awareness and improving intervention and preventive policies can reduce the incidence of brucellosis in high-risk areas.[22]With emergence of the new technologies in the field of health, Geographical Information Systems (GIS) have demonstrated the capability to discover the new epidemic pattern and hotspot areas to precisely provide policy guidance for brucellosis control[23] and significant reduction associated with the costs, care and prevention programs based on the geographical data[24]. Using the data from GIS in epidemiological investigations into the prevalence of brucellosis in Iran, Shirzadi et.al (2021) identified the high-priority areas to implement the targeted interventions to control the disease[25]. According to Azizi et.al (2019), because of traditional consumption habits of unpasteurized fresh and local dairy products, Neyshabur stands out as a highly polluted and susceptible region to brucellosis, considering the pattern of livestock distribution, the spatial distribution of this disease varies in villages[26].

In the present study, we employed a spatiotemporal distribution model to analyze local surveillance data on human brucellosis. Moreover, we focus on investigating the human and spatiotemporal distribution characteristics, as well as correlations of human brucellosis epidemics in Neyshabur, the second-largest city of Razavi Khorasan Province in the Northeast of Iran, between 2015 and 2022.

Materials and methods

Study area

This cross-sectional study was conducted in Neyshabur, the north-eastern region of Iran, with a population of 451,780 [27]. Neyshabur with a 5.653 km^2 area, located in 36° 25′ 26″ N, 58° 37′ 31″ E[28], is the second-largest city in the Khorasan Razavi province and the third-largest city in Eastern Iran. Neyshabur has 418 habitable villages organized into census blocks, which are the smallest spatial units in the cities of Iran. For this study, rural districts, a political division in Iran, were utilized as the geographical scale for conducting the spatial analyses.

Data sources and patients

The data was collected from two sources comprising both non-spatial and spatial. Firstly, the data on human brucellosis cases was obtained from the Center for Prevention and Control of Contagious Diseases, under the supervision of the Vice-Chancellor for Health of Neyshabur University of Medical Sciences from 2015 to 2022. The criteria for diagnosing patients with

human brucellosis were a positive Wright and 2-mercaptoethanol (2ME) tests in the population suspected of being infected. Secondly, the spatial divisions map of Neyshabur area was acquired through the municipal authorities. Because the lack of registered latitude and longitude for human brucellosis cases, we had to geocode patients' addresses using Google My Maps. To ensure patient privacy, we used the geomasked technique. Each patient's address was randomly assigned within a radius of half a kilometer around his/her home. Because of the study design that was executed at the district level, the probability pointing address of patients did not limited our study. All maps and spatial analyses in this study were created by the authors using ArcGIS software, version 10.8.2.

Ethics approval and consent to participate

This retrospective study, approved by the Research Ethical Committee of Neyshabur University of Medical Sciences under the ethical code IR.NUMS.REC.1401.042, is in compliance with ethical standards. After the conformation of the study, the data related to human participants received on April 9, 2023 was received by the study team from the Center for Prevention and Control of Contagious Diseases in a as blinded format. This means that data were de-identified at the source by trained personnel. Additionally, the study team is committed to using these data only for the present study, and no one outside the study team has access to the data.

Anselin Local Moran's I

Anselin's Local Moran I is a local spatial autocorrelation measure derived from the global Moran's I statistic. It calculates the Moran's I value for each individual spatial unit (e.g., district) by creating a neighborhood around that unit and comparing its value to the overall study area[29]. in this study, the spatial statistic was applied in this study to identify the high and low incidence districts of Neyshabur considering human brucellosis incidence between 2015 to 2022. The output of Anselin's Local Moran I analysis is a new map that classifies the districts of Neyshabur into five classes based on the spatial patterns of human brucellosis incidence (High-High cluster, Low-Low cluster, High-Low cluster, Low-High cluster, not cluster).

Global Moran's I

Global Moran's I is a spatial autocorrelation statistic that simultaneously measures spatial autocorrelation in a study area based on the attribute values of interest [29]. In this study, we used Global Moran's I to assess the distribution pattern of human brucellosis incidence in Neyshabur between 2015 and 2022. The null hypothesis states that the spatial distribution of human brucellosis incidence in each year is random. The interpretation of Global Moran's I is based on the P-value and Z-score. The null hypothesis may be rejected if the P-value is statistically significant ($P < 0.05$). If p-value is statistically significant, and the z-score is positive, it means that the high and/or low values of human brucellosis incidence in the dataset is more spatially clustered than would be expected. conversely, if the p-value is statistically significant, and the z-score is negative, it means that the spatial distribution of high values and low values of human brucellosis

incidence in the dataset is more spatially dispersed than would be expected. [30].

Results

Between 2015 and 2022, a total of 4,568 cases of human brucellosis were reported in Neyshabur. The average incidence rate over these eight years was 108.26 cases per 100,000 people. The annual incidence rates indicate an increasing trend in this disease from 2018 to 2022, while no consistent trend was observed in other years. Throughout the study period, men were diagnosed with brucellosis, 55.36% more than women. Additionally, 89.32% of the patients were rural residents. The highest incidence rates were observed in the summer and spring, with 34.76% and 33.84% of cases, respectively. See Table 1 for more details.

Table 1The characteristics of Seasonally incidence of Brucellosis cases in Neyshabur from 2015-2022

Year \ variable	Cases	IR	Gender (% of males)	Location (% of rural)	Season (% of patients)			
					Spring	Summer	Autumn	Winter
2015	329	64.27	60.49	83.59	38.90	35.26	13.07	12.77
2016	274	53.72	56.93	91.24	39.78	43.07	12.41	4.74
2017	292	56.95	57.19	95.21	39.04	36.30	16.44	8.22
2018	233	46.30	54.94	93.56	31.33	40.77	13.73	14.16
2019	559	109.32	58.32	91.41	20.39	37.92	16.46	25.22
2020	640	122.21	56.56	92.81	33.91	48.75	15.47	1.88
2021	1356	251.94	52.95	92.12	36.95	27.58	25.52	9.96
2022	885	161.38	53.45	79.89	32.77	28.81	17.74	20.68

Total	4568	86.79 (54.53 -151.59) [b]	55.36	89.32	33.84	34.76	18.63	12.76

a= Incidence Rate (IR) per 100,000

b= Median (IQR)

The median age of patients is 38 years, with an interquartile range of 25 to 52 years. A significant relationship between age and gender was observed ($P < 0.001$). See Figure 1.

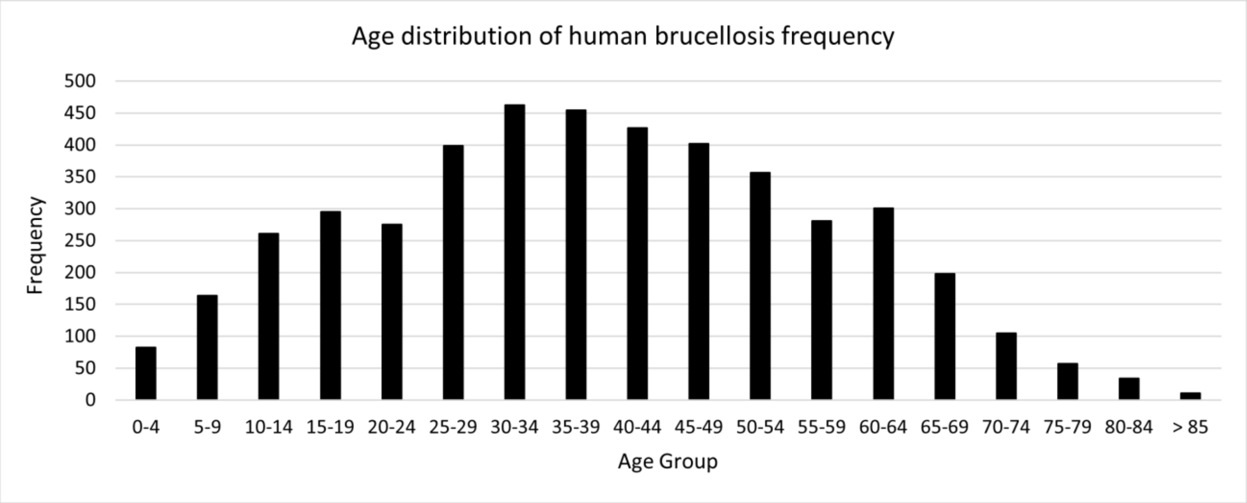

Figure 1 Age distribution of human brucellosis cases in the Neyshabur, Iran during 2015-2022

Monthly incidence data for brucellosis reveal that the lowest rates of infection occur in December, while the highest rates are observed in June. An increasing trend in incidence is noted from December to June, followed by a decreasing trend from June to December. See Figure 2

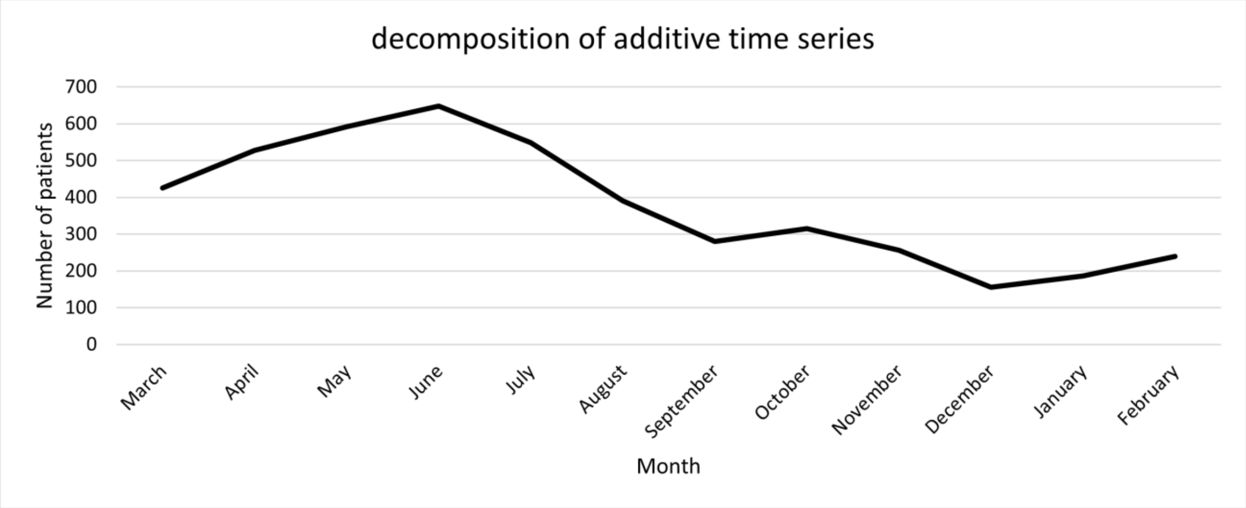

Figure 2 The monthly distribution of human brucellosis cases in the Neyshabur, Iran during 2015-2022

According to self-reported data from infected patients during the study period in Neyshabur, 84.60% had direct contact with animals at work or home. Moreover, 64.30% of patients had a history of consuming unpasteurized dairy products, this indicates indirect contact. Among those who had direct or indirect contact with animals, 38.40% were aware that animal vaccination against brucellosis had been performed. Furthermore, 16.70% of patients reported that other family members also were suffered from the disease. See Table 2

Table 2 Distribution of the relative frequencies of the population with brucellosis based on the studied variables

Variable / Year	Direct interaction with livestock			Consuming raw dairy products			Livestock vaccination			Household member infections		
	Yes	No	Unclear	Yes	No	Unclear	Yes	No	Unclear	Yes	No	Unclear
2015	91.2	7.5	1.2	69.0	25.8	5.2	24.0	30.4	45.6	18.8	73.3	7.9
2016	90.9	6.5	2.6	66.8	25.5	7.7	18.6	12.8	68.6	20.8	69.0	10.2
2017	94.5	4.5	1.0	71.2	21.3	7.5	18.5	37.3	44.2	13.7	75.3	11.0
2018	91.4	6.0	2.6	80.3	12.0	7.7	29.2	38.6	32.2	14.6	73.0	12.4
2019	90.7	8.6	0.7	69.6	28.4	2.0	37.7	49.4	12.9	17.5	73.0	9.5

2020	93.3	6.7	0.0	56.6	43.4	0.0	37.8	47.4	14.8	22.0	76.6	1.4
2021	89.8	9.7	0.5	57.6	34.8	7.6	43.1	33.1	23.8	16.7	73.2	10.1
2022	57.4	38.1	4.5	67.7	28.7	3.6	52.6	38.6	8.8	7.9	85.5	6.6
Total	84.7	13.7	1.6	64.3	30.8	4.9	38.4	37.3	24.3	16.0	75.9	8.1

The geographical distribution of disease cases indicates that most infections were concentrated in the southern and southeastern areas of Neyshabur during the study period. See Figure 3.

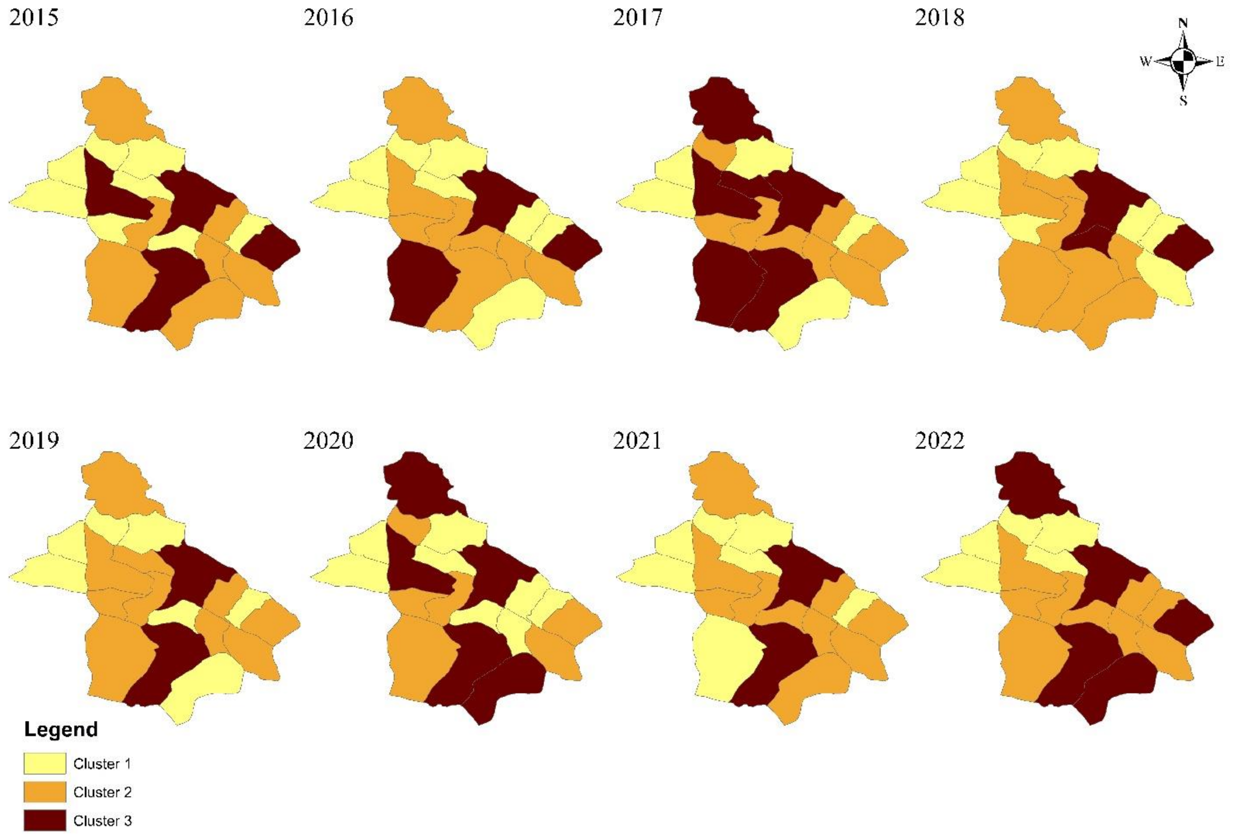

Figure 3 Geographical distribution of human brucellosis cases at district level in Neyshabur, Iran during 2015-2022. This figure was created by the authors using ArcGIS software, version 10.8.2.

The results of Moran's Local Indicator of Spatial Association (LISA) test for the districts of Neyshabur reveal that, in six out of the eight years studied (excluding 2016 and 2017), Cold Spot clusters were more frequently found in the northern areas of the city. Conversely, Hot Spot clusters were observed in the southwestern region during 2017 and 2019. Additionally, Low-High Outlier clusters were noted in the southeastern region during 2017, 2019, and 2021. See Figure 4

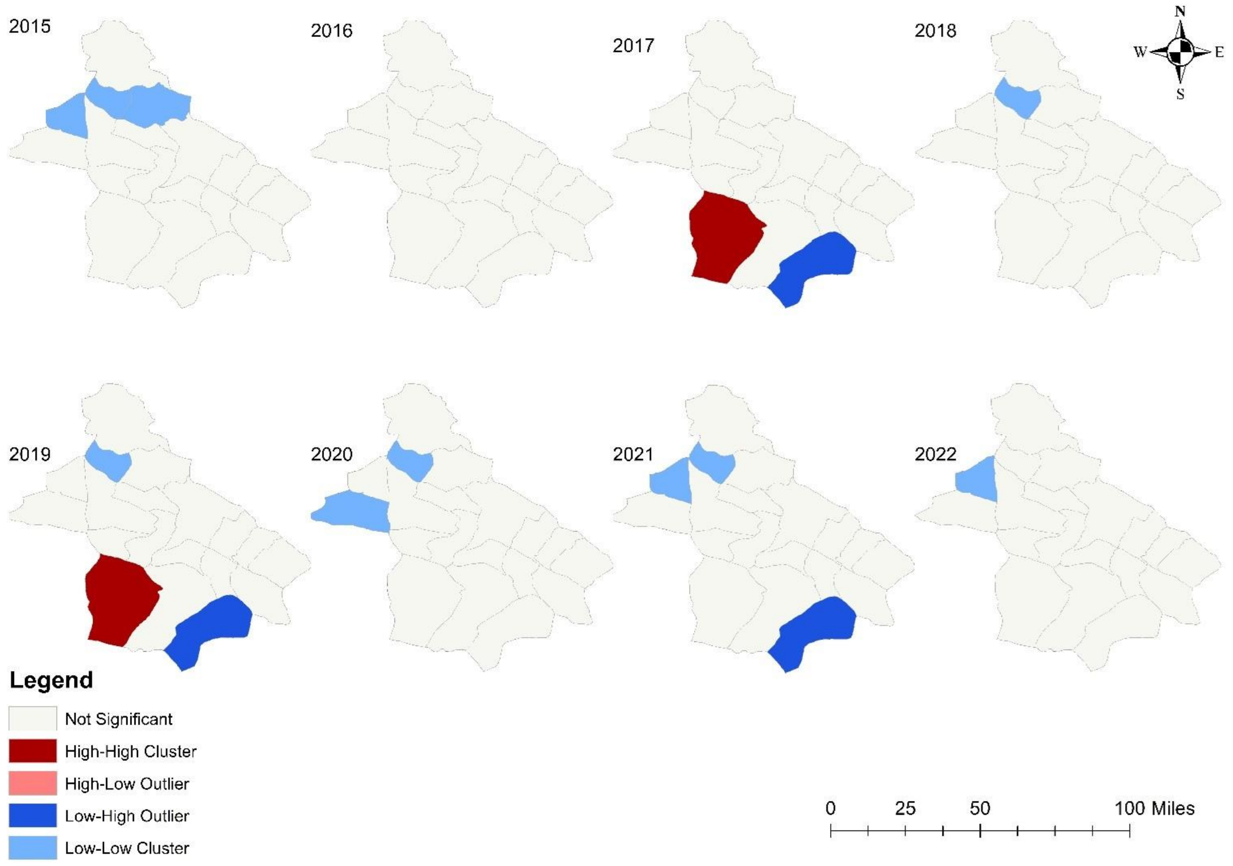

Figure 4 Spatial cluster analysis of human brucellosis in district level of Neyshabur, Iran during 2015-2022. This figure was created by the authors using ArcGIS software, version 10.8.2.

The results of the global Moran's I spatial test, used to determine the geographical distribution of human brucellosis in Neyshabur from 2015 to

2022, showed that the Moran's I index ranged from 0.0123 to 0.2860. A Z-score of less than 1.96 and P-values greater than 0.05 indicate that the geographic distribution of human brucellosis in Neyshabur was random during the study period. See Table 3

Year	Moran's I	Variance	Z score	P value	Aggregation
2015	0.184453	0.048362	1.091377	0.275107	No
2016	0.110425	0.048846	0.751000	0.452653	No
2017	0.012333	0.050060	0.303427	0.761565	No
2018	0.055398	0.047653	0.508272	0.611263	No
2019	0.240794	0.044657	1.402355	0.160809	No
2020	0.245693	0.049658	1.351850	0.176423	No
2021	0.146019	0.048417	0.916091	0.359619	No
2022	0.286051	0.049117	1.541381	0.123224	No

Discussion

During 2015 -2022, a total of 4,568 human brucellosis cases were reported in Neyshabur, with an annual incidence rate 108.26 per 100,000 inhabitants. Although this is likely underestimated as it only includes patients who visited the health centers of University in Neyshabur and doesn't account for those who sought treatment at private clinics. In addition, since the general signs and symptoms of brucellosis (e.g. fever, shivering, sweating, weakness, fatigue, headache, lumbar pain and etc.) are vague and similar to influenza, it may lead to delays in the early diagnosis of disease and doing diagnostic tests[31]. Even considering undiagnosed cases, the incidence rate of the disease in Neyshabur is higher than the average for Razavi Khorasan Province. Base on the results

conducted by Norouzinezhad, the incidence rate of brucellosis in Razavi Khorasan Province was reported 35.17, 29.52, and 39.30 in 2015, 2016, and 2017, respectively, while during the same years, the incidence rate in Neyshabur was 64.27, 53.72, and 56.95, respectively [11]. Regarding the rising trend in the incidence rate in Neyshabur from 2018 to 2022 indicates a clear need for more detailed planning to control the disease in the coming years.

Our findings showed that the ratio of rural to urban residents affected by brucellosis is approximately 9 to 1. Consistent with previous studies, the rural population is at a higher risk of contracting the disease [11, 25, 32, 33]. This difference in infection rates may be associated with the rural lifestyle in Iran, where most people are engaged in agriculture and animal husbandry. Nevertheless, a study conducted in Semnan Province found that 70.9% of patients lived in urban areas [34]. Norouzinezhad identified brucellosis as an occupational disease [11]. It can justify our findings that indicates the lack of a significant difference in brucellosis incidence between genders (with a ratio of men to women at 1.24), because, in rural areas, women are involved in animal husbandry similar to men and take responsibilities such as cleaning pens and milking [35]. The average age of men affected by brucellosis is 35.68 years, compared to 42.37 years for women. This notable difference may be due to men entering animal husbandry at a younger age.

Most studies have shown direct contact with livestock and consumption of raw dairy products as risk factors for brucellosis. Consistent with our findings, several studies have also highlighted direct contact with livestock as the most prevalent risk factor for the disease in humans [12, 25]. Almost 85% of patients

reported that they are in direct contact with animals at their workplace or living place. Given that 90% of rural residents affected by brucellosis contacting to animals, this exposure can be seen as a key risk factor. Additionally, our findings reveal that about 65% of patients had a history of consuming raw dairy products. Previous studies reported raw dairy consumption as the most common risk factor for brucellosis, which differs from our results [18, 36, 37] In rural areas, there is a common belief that unprocessed dairy products are more nutritious than pasteurized ones. This perception increases the preference for consuming raw dairy products, such as milk, yogurt, and cream [11]. Educating programs, particularly among women who are more involved in family nutrition, could enhance awareness and reduce the consumption of raw dairy products. Our findings indicate peak incidence between March and June, with a subsequent decrease until reaching its lowest level in December. The infection rate then rises again, reaches its peak in June. Similar studies conducted in Isfahan Province [33], Bejestan and Gonabad in Razavi Khorasan Province [32], and Zanjan Province [38] also reported the highest incidence of brucellosis in June and July. This seasonal increase is likely associated with the breeding season. During this period, contact with fetal secretions during and after childbirth, combined with the increased production and consumption of unpasteurized milk and dairy products in summer, contributes to a higher incidence of brucellosis [11, 36, 39]. To mitigate this, educational programs focusing on protective measures against contact with livestock and their secretions, as well as reducing the consumption of raw dairy products during the summer months, could be effective in preventing brucellosis.

The spatial clustering analysis of brucellosis incidence across 19 districts in Neyshabur from 2015 to 2022 revealed spatial clusters in all years except 2016. The distribution of these clusters was consistent over the years, with high incidence rates often observed in the southwestern part of the city. This region is characterized by poorer and more isolated villages, which often lack adequate health infrastructure and monitoring, particularly considering livestock exchange between neighboring villages. This shows insufficient attention to livestock immunization, inadequate quarantine actions in villages with high brucellosis incidence, and a lack of effective monitoring of public health in these areas. Additionally, clusters with low incidence have consistently been observed in the northern and northwestern regions over the years. The lower incidence of brucellosis in these areas is likely due to the low population density in the villages. This region, situated between the cities of Quchan and Khoshab in Razavi Khorasan Province, is known for its small population compared to other villages. Between 2017 and 2019, as well as in 2021, the Belherat district, located near high-incidence areas, was characterized by a low incidence of the disease. A limitation of our study is the inability to determine the reasons for this spatial variation in the southeastern part of Neyshabur during these years.

The results of Moran's spatial Autocorrelation (Global Moran's I) indicate that over the eight years studied, the distribution of malaria in Neyshabur's villages was random, with no consistent pattern of high or low incidence. This suggests that there is a need for more comprehensive and precise studies to better predict brucellosis incidence in Neyshabur City, especially given the rising trend of

the disease. Enhanced and detailed research is necessary to address the increasing rates of brucellosis in this city.

Conclusion

The findings of this study can inform the planning of educational initiatives, particularly those targeting mothers, as well as strategies for animal vaccination. These efforts should be emphasized during the months leading up to the peak human brucellosis season and in regions with high incidence rates. Future research should explore the extent of livestock vaccination coverage and its correlation with the incidence of human brucellosis.

Limitations & Strength

Due to inadequate accurate registration of the livestock numbers by districts, the lack of access to local animal vaccination data based on the geographical distribution of livestock husbandry, and the deficiency in measuring the annual rainfall at the district level in Neyshabur, the present study didn’t examine the effect of these factors on the annual incidence rate of human brucellosis. Despite these limitations, we indicated the spatial pattern of human brucellosis incidence in Neyshabur and increased our knowledge about the most common causes of the disease in the human population. This information can be used as a scientific resource for revising prevalence strategies to control or eradicate human brucellosis in Neyshabur.

Acknowledgments

We would like to extend our sincere gratitude to Dr. Benyamin Hosseini for his valuable comments and suggestions that allowed us to improve the quality of the study. Additionally, we are especially grateful for the help of Dr. Ensiyeh Mollanoroozy for her expert editing support, who spent hours to strengthen the overall quality of the manuscript beyond correcting grammatical and spelling mistakes.

Funding

This study was supported by the Student Research Committee (SRC) funds of Neyshabur University of Medical Sciences (NUMS). The funders had no role in study design, data collection and analysis, decision to publish, or preparation of the manuscript.

References

1. Nicoletti P. Brucellosis: past, present and future. Prilozi. 2010;31(1):21-32.
2. Gwida M, Al Dahouk S, Melzer F, Rösler U, Neubauer H, Tomaso H. Brucellosis - regionally emerging zoonotic disease? Croat Med J. 2010;51(4):289-95.
3. Mirambo MM, Mgode GF, Malima ZO, John M, Mngumi EB, Mhamphi GG, Mshana SE. Seropositivity of Brucella spp. and Leptospira spp. antibodies among abattoir workers and meat vendors in the city of Mwanza, Tanzania: A call for one health approach control strategies. PLoS Negl Trop Dis. 2018;12(6):e0006600.
4. Dadar M, Shahali Y, Whatmore AM. Human brucellosis caused by raw dairy products: A review on the occurrence, major risk factors and prevention. Int J Food Microbiol. 2019;292:39-47.

5. Esmaeili H. Brucellosis in Islamic republic of Iran. Journal of medical bacteriology. 2014;3(3-4):47-57.
6. Hasanjani Roushan MR, Ebrahimpour S. Human brucellosis: An overview. Caspian J Intern Med. 2015;6(1):46-7.
7. Mirnejad R, Jazi FM, Mostafaei S, Sedighi M. Epidemiology of brucellosis in Iran: A comprehensive systematic review and meta-analysis study. Microb Pathog. 2017;109:239-47.
8. Soleimani A, Alizadeh S, Farshad MS, Kusha A, Mohamdzadeh M, Haghiri L, et al. Descriptive epidemiology of human brucellosis in east Azerbaijan, 2001-2009. Medical Journal of Tabriz University of Medical Sciences. 2012;34(1):63-9.
9. Mohammadian M, Mohammadian-Hafshejani A. Epidemiological characteristics and incidence rate of brucellosis over a period of 14 years in the Tiran-Karvan Township, Isfahan, Iran. Journal of Isfahan Medical School. 2014;32(293):1103-9.
10. Mostafavi E, Asmand M. Trend of brucellosis in Iran from 1991 to 2008. 2012.
11. Norouzinezhad F, Erfani H, Norouzinejad A, Ghaffari F, Kaveh F. Epidemiological Characteristics and Trend in the Incidence of Human Brucellosis in Iran from 2009 to 2017. J Res Health Sci. 2021;21(4):e00535.
12. Norouzinezhad F, Erfani H, Norouzinejad A, Kaveh F, Ghaffari F. Epidemiological indices and trend of incidence of human brucellosis in Khorasan-e-Razavi Province from 2009 to 2016. Journal of Military Medicine. 2019;21(4):362-71.
13. Havas KA, Ramishvili M, Navdarashvili A, Hill AE, Tsanava S, Imnadze P, Salman MD. Risk factors associated with human brucellosis in the country of Georgia: a case-control study. Epidemiol Infect. 2013;141(1):45-53.
14. Franc KA, Krecek RC, Häsler BN, Arenas-Gamboa AM. Brucellosis remains a neglected disease in the developing world: a call for interdisciplinary action. BMC Public Health. 2018;18(1):125.
15. Babaie E, Alesheikh AA, Tabasi M. Spatial prediction of human brucellosis (HB) using a GIS-based adaptive neuro-fuzzy inference system (ANFIS). Acta Trop. 2021;220:105951.
16. Bosilkovski M, Edwards MS, Calderwood S. Brucellosis: epidemiology, microbiology, clinical manifestations, and diagnosis. Uptodate, United States. 2019.
17. Khalid HM. Seroprevalence and Associated Risk Factors of Brucellosis Among Human Population in Duhok City, Iraq. Infect Drug Resist. 2023;16:2805-11.
18. Zeinali M, Doosti S, Amiri B, Gouya MM, Godwin GN. Trends in the Epidemiology of Brucellosis Cases in Iran during the Last Decade. Iran J Public Health. 2022;51(12):2791-8.
19. wikipedia. Counties_of_Iran [Available from: https://en.wikipedia.org/wiki/Counties_of_Iran.
20. Hashemi SH, Gachkar L, Keramat F, Mamani M, Hajilooi M, Janbakhsh A, et al. Comparison of doxycycline-streptomycin, doxycycline-rifampin, and ofloxacin-rifampin in the treatment of brucellosis: a randomized clinical trial. Int J Infect Dis. 2012;16(4):e247-51.
21. Sofian M, Aghakhani A, Velayati AA, Banifazl M, Eslamifar A, Ramezani A. Risk factors for human brucellosis in Iran: a case-control study. Int J Infect Dis. 2008;12(2):157-61.
22. Bat-Erdene D, Chuang YC, Chuang KY. Brucellosis knowledge and preventive practices among herders in Western Mongolia. Zoonoses Public Health. 2019;66(1):133-9.
23. Tao Z, Chen Q, Chen Y, Li Y, Mu D, Yang H, Yin W. Epidemiological Characteristics of Human Brucellosis - China, 2016-2019. China CDC Wkly. 2021;3(6):114-9.
24. Halimi L, Bagheri N, Hoseini B, Hashtarkhani S, Goshayeshi L, Kiani B. Spatial analysis of colorectal cancer incidence in Hamadan Province, Iran: a retrospective cross-sectional study. Applied Spatial Analysis and Policy. 2020;13:293-303.
25. Shirzadi MR, Mohammadi P, Moradi G, Goodarzi E, Khazaei S, Moayed L, Khazaei Z. The Incidence and Geographical Distribution of Brucellosis in Iran Using Geographic Information System and Prediction of its Incidence in 2021. J Prev Med Hyg. 2021;62(3):E635-e4.

26. Azizi Mayvan F, Hashtarkhani S, Latifi M, Darrudi R, Hoseini B. Geographic disparities of brucellosis distribution in Neyshabur between 2014 and 2016: a cross-sectional study based on geographical information systems. J Neyshabur Univ Med Sci. 2019;7(4):53-64.
27. Census of the Islamic Republic of Iran, 1395 (2016)". AMAR (in Persian). The Statistical Center of Iran 2016 [Available from: https://web.archive.org/web/20220402220537/https://www.amar.org.ir/.
28. contributors O. Nishapur County 2023 [Available from: https://geohack.toolforge.org/geohack.php?pagename=Nishapur_County&params=36_25_26_N_58_37_31_E_dim:60km_type:city(451780)_region:IR-09.
29. Anselin L. Local indicators of spatial association—LISA. Geographical analysis. 1995;27(2):93-115.
30. Amsalu ET, Akalu TY, Gelaye KA. Spatial distribution and determinants of acute respiratory infection among under-five children in Ethiopia: Ethiopian Demographic Health Survey 2016. PloS one. 2019;14(4):e0215572.
31. Bagheri H, Tapak L, Karami M, Amiri B, Cheraghi Z. Epidemiological Features of Human Brucellosis in Iran (2011-2018) and Prediction of Brucellosis with Data-Mining Models. J Res Health Sci. 2019;19(4):e00462.
32. Riabi HRA, Riabi HRA, Razmara H. Epidemiological Feature of the Human Brucellosis Prevalence in People in Southern Cities of Khorasan Razavi, Iran. Zahedan Journal of Research in Medical Sciences. 2017;19(4).
33. Salahshouri A, Ramezanpour J, Gheibipour H. Epidemiological Study of the Brucellosis in Iran, Isfahan, 2010–2015. Epidemiology and Health System Journal. 2021;8(3):95-9.
34. Kamal S, Hashemi S, Nasaji M, Moshiri E, Shahriyari R, Azizi A. Frequency of reported cases of brucellosis to province health center from public and private sectors in Semnan 2006-2007. 2009.
35. Pakzad R, Pakzad I, Safiri S, Shirzadi MR, Mohammadpour M, Behroozi A, et al. Spatiotemporal analysis of brucellosis incidence in Iran from 2011 to 2014 using GIS. Int J Infect Dis. 2018;67:129-36.
36. Pordanjani SR, Atamaleki A, Amiri M, Khazaei Z, Fallahzadeh H, Alayi R, et al. Study on epidemiological status, spatial and temporal distribution of human brucellosis in kohgiluyeh and Boyer-Ahmad Province during 2011–2017. Advances in Human Biology. 2020;10(1):22-8.
37. Dehghan A, Sadeghian M, Jafarnejad A. Epidemiologic survey of Brucellosis in Fasa during 2009-2017. medical journal of mashhad university of medical sciences. 2019;62(2):1449-55.
38. Rostami Z, Faghihzadeh S, Tagilou B, Khosravi Y. Determining the Incidence Rate and Risk Factors of Brucellosis in Zanjan Province (Iran) from 2012 to 2017: A Spatiotemporal Analysis. 2020.
39. Moosazadeh M, Abedi G, Kheradmand M, Safiri S, Nikaeen R. Seasonal pattern of brucellosis in Iran: A systematic review and meta-analysis. 2016.

Click here to access/download
Supporting Information
Data Availability Statement.docx

Figures

Click here to access/download
Supporting Information - Compressed/ZIP File Archive
Figures.rar

Spatio-temporal analysis of human brucellosis distribution in Neyshabur, Iran from 2015 to 2022, a cross-sectional study

Ayoub Fathabadi[1¶], Fatemeh Khorashadizadeh[2&], Reza Darrudi[1&], Mohammad Maroosi[3*]

[1] Department of Health Information Technology, Faculty of Health and Paramedicine,

Neyshabur University of Medical Sciences, Neyshabur, Iran

[2] Department of Epidemiology and Biostatistics,

Neyshabur University of Medical Sciences, Neyshabur, Iran

[3] Department of Environmental Health Engineering, Faculty of Health and Paramedicine, Neyshabur University of Medical Sciences, Neyshabur, Iran

* Corresponding author
E-mail: maroosim@nums.ac.ir

Abstract

Background: Human brucellosis is a global endemic disease and a significant public health concern. This cross-sectional study aims to explore the characteristics of human brucellosis, as well as its spatial and temporal clustering.

Methods: ~~The~~ this study with ethical code IR.NUMS.REC.1401.042, analyzed the population infected with human brucellosis in Neyshabur, a city in the east of Iran from 2015 to 2022.~~data on human brucellosis was collected~~ Patient data were obtained from the Center for Prevention and Control of Contagious Diseases.~~,~~ ~~under the supervision of the Vice-Chancellor for Health of Neyshabur University of Medical Sciences from 2015 to 2022.~~ Spatial ~~autocorrelation~~ analysis was conducted using Anselin Local Moran's I with Arc GIS 10.8.2 and Global Moran's I with Arc GIS Pro 3.0.1. ~~and statistical~~ Descriptive statistical analysis~~-~~ were performed using SPSS 26.~~employed to identify trends in changes in the spatial and temporal distribution and human characteristics of brucellosis~~.

Results: During the study period, a total of 4568 brucellosis patients were reported. The incidence of disease was higher in men with 55.37%. The majority of patients belonged to the 30 to 39-year-old age group. Temporal clustering analysis revealed the peak incidence between March and June. Spatial clustering analysis indicated a higher prevalence in the southern and southeast districts of Neyshabur. The results of Global Moran's I analysis indicated that the distribution of brucellosis was not clustered in Neyshabur.

Conclusion:

The findings of this study highlight the importance of educational programs for mothers and animal vaccination strategies, particularly in the months leading up to the peak brucellosis season in high-incidence areas. Further research is needed to assess the coverage of livestock vaccination and its impact on human brucellosis rates

It is crucial to understand the seasonal trends in brucellosis to implement effective prevention strategies. By analyzing the data, our study shows a high infection prevalence from March to July between 2015 and 2022 due to contact with infected livestock. Given the increased prevalence, the emphasis is on preventive strategies including livestock vaccination, and educating families and farmers are needed to mitigate the risk.

Key words: Human brucellosis, Spatio-Temporal Analysis, Spatial Analysis, Cluster Analysis, Space-Time Clustering, Geographic Information Systems

Introduction

Human Brucellosis is one of the seven neglected and under-detected acute febrile diseases that remains one of the major causes of morbidity throughout the world[1]. It results from the transmission of the brucellosis bacterial agent from infected animals to humans [2]. In populations with the traditional lifestyles, inhalation, direct contact with animal and consumption of unpasteurized dairy products and undercooked meat products are the primary modes transmissions leading to human brucellosis[3, 4]. Human brucellosis is endemic in Iran were many people, especially those in rural areas, commonly keep livestock at their residence [5] This traditional lifestyle significantly contributes to the spread of brucellosis within the Iranian population [6]. The incidence of human brucellosis in Iran ranges from 7 to 276 per 100,000 people [7]. Based on previous research, Iran ranks fourth in the world in terms of incidence rate of human brucellosis [8-10] Although the highest incidences rates of human brucellosis are related to the west and northwest regions of the Iran [7] the most frequent cases of human brucellosis pertain to Razavi Khorasan, the second most populous province in northeast of Iran [11]. According to Norouzinezhad, the incidence of human

brucellosis was more than 20 per 100,000 people in Razavi Khorasan between 2009-2016[12] Research has established that brucellosis as a bacterial infection, transmitted to human from ingestion of contaminated dairy or contact with infected animals, is an important risk factor in human health. This relates to occupational, especially working with animals, ethnic and regional aspects of individuals' lifestyle. There is now a strong body of evidence to support that brucellosis can cause a reduction in quality of life[13], herd productivity, and food security[14] Considering human health, this disease affects the body's immune system leading to flu-like symptoms such as fever, weakness, malaise, weight loss and fatigue[15]. The medical, veterinary and socioeconomic implications are emphasized as the other significant public health concerns associated with brucellosis[15]. Brucellosis prevalence has been reported to be highest in some areas such as Central Asia, Mediterranean countries, Latin America, and sub-Saharan Africa. Due to the increasing trends in international traveling, trade and migration, the prevalence of the disease has increased in all ages of the population[16]. The confirmed cases are estimated in studies in various Asian countries with variable prevalence, including India (16.7%), Turkey (8.8%), Saudi Arabia (12.7%), Iraq (12.6%), and Iran (6.59%)[17]. However, a significant reduction in the incidence of cases has been observed in some countries, including Australia, New Zealand, and Japan[14]. This suggests that the prevention or even elimination of human brucellosis may be feasible which requires the prevention programs to achieve the desired outcomes. Iran, also, is endemic for human brucellosis, notably prevalent in areas where people reside in close contact with infected animals or animal products.[10, 18]. According to the Iranian Ministry of Health (MOH), Razavi Khorasan province, with an average of 21–30 per 100,000 people annually, is among the provinces with the highest incidence rate of human brucellosis in Iran [18]. Neyshabur, the second-largest city in the province, has over350 villages [19]. where traditional livestock breeding is common. This increases

risk of infection for all members of family due to direct contact with contaminated domestic animals. Further, prevalence of brucellosis varies by time and place[20, 21]. Between 2009 and 2016, the incidence of human brucellosis in Neyshabur ranged from 25.66 to 73.04 cases per 100,000 people [12]. increasing public awareness and improving intervention and preventive policies can reduce the incidence of brucellosis in high-risk areas.[22] By 2017, the annual disease rate was estimated at one case per 100,000 people that the highest rate was reported in the western and northern regions of the country[7]. With emergence of the new technologies in the field of health, Geographical Information Systems (GIS) have demonstrated the capability to discover the new epidemic pattern and hotspot areas to precisely provide policy guidance for brucellosis control[23] and significant reduction associated with the costs, care and prevention programs based on the geographical data[24]. Using the data from GIS in epidemiological investigations into the prevalence of brucellosis in Iran, Shirzadi et.al (2021) identified the high-priority areas to implement the targeted interventions to control the disease[25]. According to Azizi et.al (2019), because of traditional consumption habits of unpasteurized fresh and local dairy products, Neyshabur stands out as a highly polluted and susceptible region to brucellosis, considering the pattern of livestock distribution, the spatial distribution of this disease varies in villages[26].

In the present study, we employed a spatiotemporal distribution model to analyze local surveillance data on human brucellosis. Moreover, we focus on investigating the human and spatiotemporal distribution characteristics, as well as correlations of human brucellosis epidemics in Neyshabur, the second-largest city of Razavi Khorasan Province in the Northeast of Iran, between 2015 and 2022.

Materials and methods

Study area

This cross-sectional study was conducted in Neyshabur, the north-eastern region of Iran, with a population of 451,780 [27]. Neyshabur with a 5.653 km^2 area, located in 36° 25′ 26″ N, 58° 37′ 31″ E[28], is the second-largest city in the Khorasan Razavi province and the third-largest city in Eastern Iran. Neyshabur has 418 habitable villages organized into census blocks, which are the smallest spatial units in the cities of Iran. For this study, rural districts, a political division in Iran, were utilized as the geographical scale for conducting the spatial analyses.

Data sources and patients

The data was collected from two sources comprising both non-spatial and spatial. Firstly, the data on human brucellosis cases was obtained from the Center for Prevention and Control of Contagious Diseases, under the supervision of the Vice-Chancellor for Health of Neyshabur University of Medical Sciences from 2015 to 2022. The criteria for diagnosing patients with human brucellosis were a positive Wright and 2-mercaptoethanol (2ME) tests in the population suspected of being infected. Secondly, the spatial divisions map of Neyshabur area was acquired through the municipal authorities. Because the lack of registered latitude and longitude for human brucellosis cases, we had to geocode patients' addresses using Google My Maps. To ensure patient privacy, we used the geomasked technique. Each patient's address was randomly assigned within a radius of half a kilometer around his/her home. Because of the study design that was executed at the district level, the probability pointing address of patients did not limited our study. All maps and spatial analyses in this study were created by the authors using ArcGIS software, version 10.8.2.

Formatted: Font: 15 pt

Ethics approval and consent to participate

This retrospective study, approved by the Research Ethical Committee of Neyshabur University of Medical Sciences under the ethical code IR.NUMS.REC.1401.042, is in compliance with ethical standards. After the conformation of the study, the data related to human participants received on April 9, 2023 was received by the study team from the Center for Prevention and Control of Contagious Diseases in a as blinded format. This means that data were de-identified at the source by trained personnel. Additionally, the study team is committed to using these data only for the present study, and no one outside the study team has access to the data.

Anselin Local Moran's I

Anselin's Local Moran I , ais a local spatial autocorrelation measure derived from the Moran's I statistic,. It calculates the Moran's I value for each individual spatial unit (e.g., district) by creating a neighborhood around that unit and comparing its value to the overall study area[29] was applied in this study, the to identify significant spatial statistic clusters and outliers of districts was applied in this to identify the exhibiting high or and low incidences districts of Neyshabur considering human brucellosis incidence between 2015 to 2022. This method calculates the Moran's I value by creating a neighborhood around each district and compares it to the overall study area. When less than 5% of the Local Moran's I values, generated from permutations, display more clustering than the original data, then the significance is determined[29]. The output of Anselin's Local Moran I analysis is a new map that classifies the districts of Neyshabur into five classes based on the spatial patterns of human brucellosis incidence (High-High cluster, Low-Low cluster, High-Low cluster, Low-High cluster, not cluster).

Moreover, whereas low values in clusters indicate cold spots, hot spots refer to high values.

Global Moran's I

Global Moran's I is a spatial autocorrelation statistic that analysis tool used to simultaneously measure spatial autocorrelation in a study area based on the attribute values of interest features[29] .In this study, It's employed we used Global Moran's I to assess the distribution pattern of human brucellosis cases incidence in Neyshabur between 2015 and 2022. in the study area to identify whether the distribution shows clustering, dispersion, or randomness. The null hypothesis states that the spatial distribution of human brucellosis incidence in each year cases is random., The interpretation of Global Moran's I is based on the P-value and Z-score. The null hypothesis may be rejected if the P-value is statistically significant ($P < 0.05$). If p-value is statistically significant, and the z-score is positive, it means that the high and/or low values of human brucellosis incidence in the dataset is more spatially clustered than would be expected. conversely, if the p-value is statistically significant, and the z-score is negative, it means that the spatial distribution of high values and low values of human brucellosis incidence in the dataset is more spatially dispersed than would be expected[30]. and this hypothesis can be rejected if the Z-score exceeds 1.96 and P-value is less than 0.05, indicating statistical significance and the presence of spatial autocorrelation [30]. Furthermore, while Moran's I value near -1.0 indicates a dissimilar cluster (dispersion), an index value near +1.0 suggests a similar cluster (clustering) of human brucellosis distribution at the level of rural districts. Otherwise, a randomized distribution is inferred.

Results

Between 2015 and 2022~~A~~ a total of 4,568 cases of human brucellosis were reported ~~registered~~ in Neyshabur ~~from 2015 to 2022.~~ The average incidence rate over these eight years was 108.26 cases per 100,000 people.~~As shown in Fig 1,~~. ~~the~~ The annual incidence rates indicate an increasing trend in this ~~frequency and trend of the~~ disease ~~reveals an ascending~~ from 2018 to 2022, while ~~the other years have~~ no constant trend~~s~~ was observed in other years. Throughout the study period, men were diagnosed with brucellosis, 55.36% more than women. Additionally, 89.32% of the patients were rural residents. The highest incidence rates were observed in the summer and spring, with 34.76% and 33.84% of cases, respectively. See Table 1 for more details. ~~Our results show that the prevalence was the highest in summer with 1588 patients (34.76%) and the lowest in the winter with 583 patients (12.76%). Moreover, the prevalence in the spring and autumn was accounted for 1546 (33.85%) and 851 (18.63%) respectively.~~

Table 1The characteristics of Seasonally incidence of Brucellosis cases in Neyshabur from 2015-2022

variable / Year	Cases	IR	Gender (% of males)	Location (% of rural)	Season (% of patients)			
					Spring	Summer	Autumn	Winter
2015	329	64.27	60.49	83.59	38.90	35.26	13.07	12.77
2016	274	53.72	56.93	91.24	39.78	43.07	12.41	4.74
2017	292	56.95	57.19	95.21	39.04	36.30	16.44	8.22
2018	233	46.30	54.94	93.56	31.33	40.77	13.73	14.16
2019	559	109.32	58.32	91.41	20.39	37.92	16.46	25.22
2020	640	122.21	56.56	92.81	33.91	48.75	15.47	1.88
2021	1356	251.94	52.95	92.12	36.95	27.58	25.52	9.96
2022	885	161.38	53.45	79.89	32.77	28.81	17.74	20.68
Total	4568	86.79 (54.53 -151.59) [b]	55.36	89.32	33.84	34.76	18.63	12.76

a= Incidence Rate (IR) per 100,000

b= Median (IQR)

Similarly, Fig 2 illustrates the seasonal trend for this disease showing an increase from December, peaking up from March to June then a declining trend in a number of patients of the total patients, The median age of patients is 38 years, with an interquartile range of 25 to 52 years. A significant relationship between age and gender was observed ($P < 0.001$). See Figure 1.

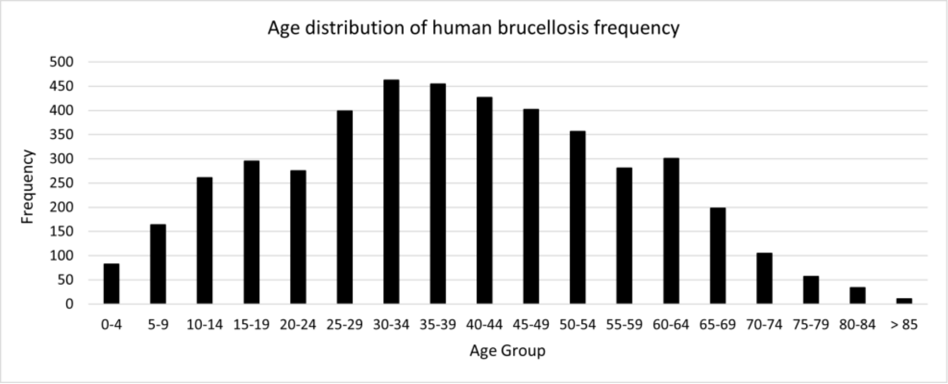

Figure 1Age distribution of human brucellosis cases in the Neyshabur, Iran during 2015-2022

Monthly incidence data for brucellosis reveal that the lowest rates of infection occur in December, while the highest rates are observed in June. An increasing trend in incidence is noted from December to June, followed by a decreasing trend from June to December. See Figure 2

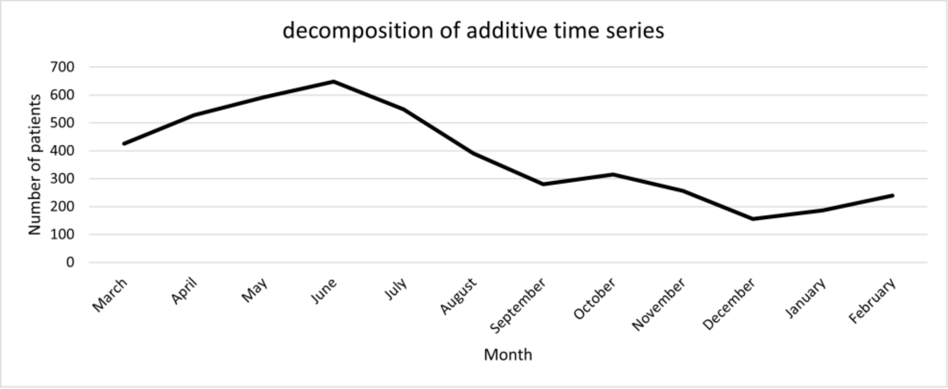

Figure 2The monthly distribution of human brucellosis cases in the Neyshabur, Iran during 2015-2022

~~88.9 % lived in rural and 11.1 % lived in urban areas. Men constituted 55.37% of the cases and women were 44.63%. The overall median age was 38 years (interquartile range (IQR)= 25-52). There was a significant relationship between patients' age and gender (*P*<0.001). Fig 3 shows distribution of patient's age. This implies that the disease affects all age groups and is not specific to any particular age group. The majority of patients fell into the 30 to 39-year-old age group~~. ~~Based on the self-reported information provided by patients, 86.01% had direct interaction with livestock at their home or work, 67.59% reported consuming raw dairy products, 50.75% were informed about the vaccination status of livestock for brucellosis, particularly those with which they had interaction or from which they had consume raw dairy products. Additionally, the study showed that the prevalence of brucellosis among the household members was17.37%.~~

According to self-reported data from infected patients during the study period in Neyshabur, 84.60% had direct contact with animals at work or home. Moreover, 64.30% of patients had a history of consuming unpasteurized dairy products, this indicates indirect contact. Among those who had direct or indirect contact with animals, 38.40% were aware that animal vaccination against brucellosis had been

performed. Furthermore, 16.70% of patients reported that other family members also were suffered from the disease. See Table 2

Table 2 Distribution of the relative frequencies of the population with brucellosis based on the studied variables

Variable / Year	Direct interaction with livestock			Consuming raw dairy products			Livestock vaccination			Household member infections		
	Yes	No	Unclear	Yes	No	Unclear	Yes	No	Unclear	Yes	No	Unclear
2015	91.2	7.5	1.2	69.0	25.8	5.2	24.0	30.4	45.6	18.8	73.3	7.9
2016	90.9	6.5	2.6	66.8	25.5	7.7	18.6	12.8	68.6	20.8	69.0	10.2
2017	94.5	4.5	1.0	71.2	21.3	7.5	18.5	37.3	44.2	13.7	75.3	11.0
2018	91.4	6.0	2.6	80.3	12.0	7.7	29.2	38.6	32.2	14.6	73.0	12.4
2019	90.7	8.6	0.7	69.6	28.4	2.0	37.7	49.4	12.9	17.5	73.0	9.5
2020	93.3	6.7	0.0	56.6	43.4	0.0	37.8	47.4	14.8	22.0	76.6	1.4
2021	89.8	9.7	0.5	57.6	34.8	7.6	43.1	33.1	23.8	16.7	73.2	10.1
2022	57.4	38.1	4.5	67.7	28.7	3.6	52.6	38.6	8.8	7.9	85.5	6.6
Total	84.7	13.7	1.6	64.3	30.8	4.9	38.4	37.3	24.3	16.0	75.9	8.1

The geographical distribution of disease cases indicates that most infections were concentrated in the southern and southeastern areas of Neyshabur during the study period. See Figure 3.

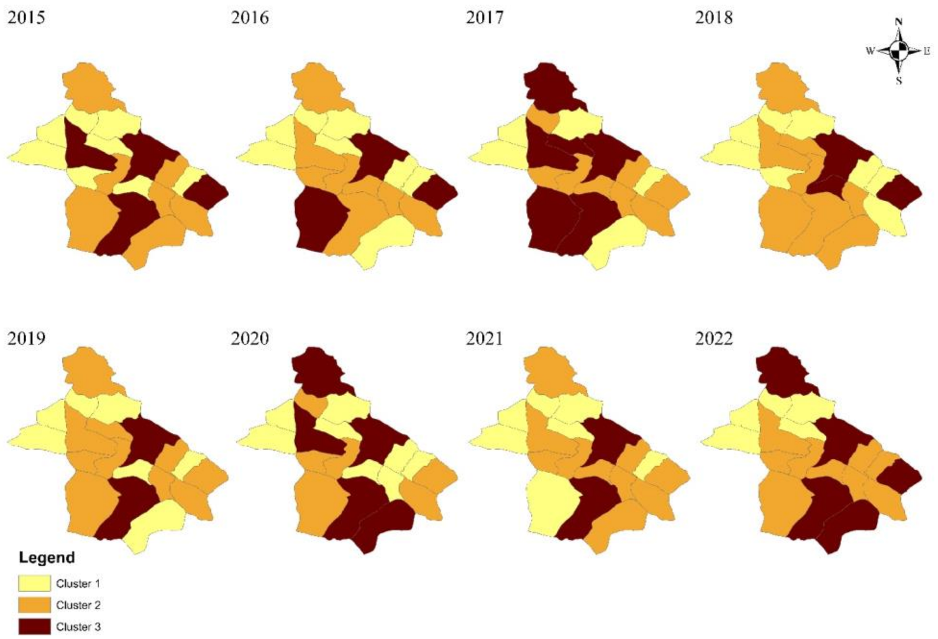

Figure 3 Geographical distribution of human brucellosis cases at district level in Neyshabur, Iran during 2015-2022~~[31]~~. This figure was created by the authors using ArcGIS software, version 10.8.2.

The results of Moran's Local Indicator of Spatial Association (LISA) test for the districts of Neyshabur reveal that, in six out of the eight years studied (excluding 2016 and 2017), Cold Spot clusters were more frequently found in the northern areas of the city. Conversely, Hot Spot clusters were observed in the southwestern region during 2017 and 2019. Additionally, Low-High Outlier clusters were noted in the southeastern region during 2017, 2019, and 2021. See Figure 4

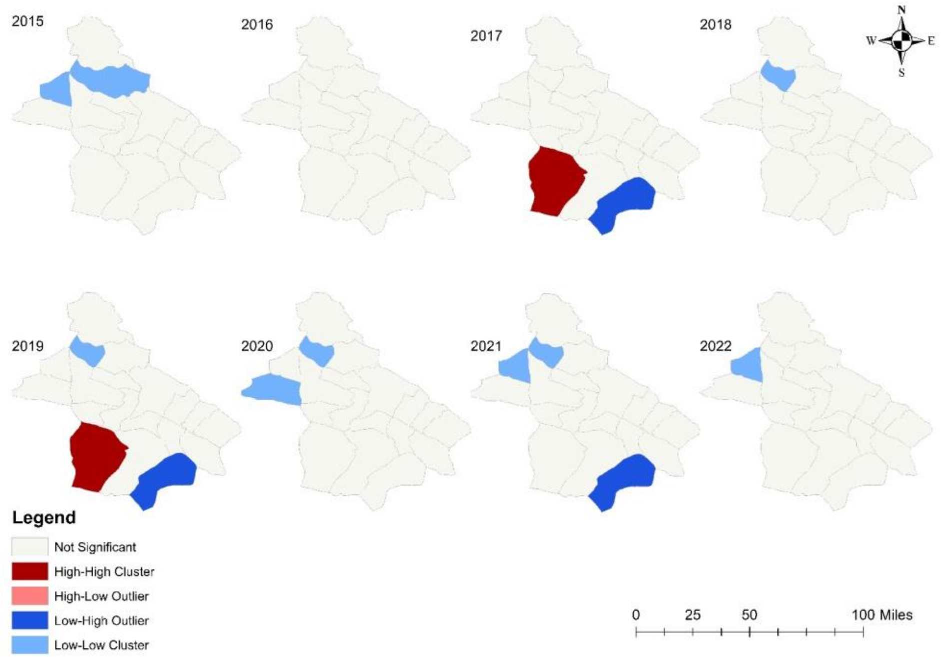

Figure 4 Spatial cluster analysis of human brucellosis in district level of Neyshabur, Iran during 2015-2022[31] This figure was created by the authors using ArcGIS software, version 10.8.2.

Fig 4 demonstrates the geographical distribution of human brucellosis. It shows a higher prevalence in the southern and southeast districts of Neyshabur. In Fig 5, the spatial statistics of Anselin's Local Moran I (cluster and outlier analysis) for human brucellosis at the district level of Neyshabur are presented. The analysis reveals that, in certain years, the northern districts exhibited significantly low-low clusters (or cold spots) of human brucellosis in the study area. Furthermore, high-high clusters (or hot spots) were observed in the southern area of the study for two years (2017-2019). Additionally, low high outliers were identified in the southern area of Neyshabur for three years (2017-2019-2021). No significant clusters or outliers were observed in other areas. Table 1 presents the calculated Moran's I value, Z score, and P value for the distribution of human brucellosis in Neyshabur from 2015 to 2022, using district

units to conduct the global autocorrelation analysis. The Moran's I value scope were 0.2737 that variable between 0.0123 to 0.2860 in during of study, respectively, the Z score values were less than 1.96 (P > .05), indicating that the incidence of human brucellosis in Neyshabur from 2015 to 2022 had a random distribution (not clustered).

The results of the global Moran's I spatial test, used to determine the geographical distribution of human brucellosis in Neyshabur from 2015 to 2022, showed that the Moran's I index ranged from 0.0123 to 0.2860. A Z-score of less than 1.96 and P-values greater than 0.05 indicate that the geographic distribution of human brucellosis in Neyshabur was random during the study period. See Table 3

Year	Moran's I	Variance	Z score	P value	Aggregation
2015	0.184453	0.048362	1.091377	0.275107	No
2016	0.110425	0.048846	0.751000	0.452653	No
2017	0.012333	0.050060	0.303427	0.761565	No
2018	0.055398	0.047653	0.508272	0.611263	No
2019	0.240794	0.044657	1.402355	0.160809	No
2020	0.245693	0.049658	1.351850	0.176423	No
2021	0.146019	0.048417	0.916091	0.359619	No
2022	0.286051	0.049117	1.541381	0.123224	No

Figure 5Global auto-correlation Moran's I value of human brucellosis in Neyshabur city, Iran during 2015 - 2022

Table 1. Global auto-correlation Moran's I value of human brucellosis in Neyshabur city, Iran during 2015 to 2022

Year	Moran's I	Variance	Z score	P value	Aggregation
2015	0.184453	0.048362	1.091377	0.275107	No
2016	0.110425	0.048846	0.751000	0.452653	No
2017	0.012333	0.050060	0.303427	0.761565	No
2018	0.055398	0.047653	0.508272	0.611263	No
2019	0.240794	0.044657	1.402355	0.160809	No
2020	0.245693	0.049658	1.351850	0.176423	No
2021	0.146019	0.048417	0.916091	0.359619	No
2022	0.286051	0.049117	1.541381	0.123224	No

Discussion

During 2015-2022, During 2015 -2022, a total of 4,568 human brucellosis cases were reported in Neyshabur, with an annual incidence rate 108.26 per 100,000 inhabitants. Although this is likely underestimated as it only includes patients who visited the health centers of University in Neyshabur and doesn't account for those who sought treatment at private clinics. In addition, since the general signs and symptoms of brucellosis (e.g. fever, shivering, sweating, weakness, fatigue, headache, lumbar pain and etc.) are vague and similar to influenza, it may lead to delays in the early diagnosis of disease and doing diagnostic tests. [32] ~~The purpose of the present study is to investigate the spatiotemporal distribution of human brucellosis in Neyshabur, using a spatial panel model to analyze the different factors affecting the prevalence of the disease in diverse geographical areas and to provide scientific strategies for the health authorities to control and prevent the disease.~~

Even considering undiagnosed cases, the incidence rate of the disease in Neyshabur is higher than the average for Razavi Khorasan Province. Base on the results conducted by Norouzinezhad, the incidence rate of brucellosis in Razavi Khorasan Province was reported 35.17, 29.52, and 39.30 in 2015, 2016, and 2017, respectively, while during the same years, the incidence rate in Neyshabur was 64.27, 53.72, and 56.95, respectively[11]. Regarding the rising trend in the incidence rate in Neyshabur from 2018 to 2022 indicates a clear need for more detailed planning to control the disease in the coming years.

Our findings showed that the ratio of rural to urban residents affected by brucellosis is approximately 9 to 1. Consistent with previous studies, the rural population is at a higher risk of contracting the disease [11, 25, 33, 34]. This difference in infection rates may be associated with the rural lifestyle in Iran, where most people are engaged in

agriculture and animal husbandry. Nevertheless, a study conducted in Semnan Province found that 70.9% of patients lived in urban areas [35]. Norouzinezhad identified brucellosis as an occupational disease[11]. It can justify our findings that indicates the lack of a significant difference in brucellosis incidence between genders (with a ratio of men to women at 1.24), because, in rural areas, women are involved in animal husbandry similar to men and take responsibilities such as cleaning pens and milking[36]. The average age of men affected by brucellosis is 35.68 years, compared to 42.37 years for women. This notable difference may be due to men entering animal husbandry at a younger age.

The results of our temporal cluster analysis demonstrated the presence of a distinct seasonality in human brucellosis. There was a seasonal trend in the prevalence of the disease with the highest rate (0.34) in summer and the lowest (0.12) in winter, reaching its peak from May to June that it can be potentially attributed to the breeding period of the animals. This result ties well with previous studies wherein the investigations into human brucellosis prevalence in different geographical areas reported the highest rate in summer[37]. However, the lowest rates were reported to vary, occurring either in autumn or winter[38]. Moreover, several studies have identified temperature as a risk factor for human brucellosis, indicating that the likelihood of incidence rises with increasing temperatures[39-41]. That aligns with our findings; the disease prevalence increases as the warmer seasons of the year (spring and summer) approach and notably decreases during the colder seasons of the year.

Considering age and gender, although the cases were not clustered in a specific age group, we found that a higher prevalence of human brucellosis among individuals in their thirties (20.09%) compared to other age brackets, with men being the most affected. This is similar to the findings of a study which were conducted to analyze

the age effect on the prevalence rates of brucellosis in different geographical areas[42]. Compared to our findings, a seroepidemiological study on 323 participants working in butcheries, slaughter rooms, and breeding farms observed the lowest rate of infection (6.3%) in 30-39 years[43]. Moreover, for gender, the majority of previous studies detected a higher positivity rate in women than males[44, 45]. The difference in disease prevalence between both genders may be associated with their engagement in working with livestock and their products. However, whether the risk for infection sexually to brucellosis is higher for males or females has not been established yet. Regarding manual milking, using its raw products, and keeping animals near the homes in most rural areas, our findings showed a rate of 88% of the human brucellosis cases. In contrast, the previous reports showed that the prevalence of the disease was 82.2% in Iran[18], 63.5% in Saudi Arabia[46], and 88.18% in China[47]. Based on these findings, we hypothesize that the rural lifestyle in Iran, characterized by the use of traditional animal husbandry and agricultural practices for the sustenance of the majority of villagers, exposes them to an increased risk.

Most studies have shown direct contact with livestock and consumption of raw dairy products as risk factors for brucellosis. Consistent with our findings, several studies have also highlighted direct contact with livestock as the most prevalent risk factor for the disease in humans[12, 25]. Almost 85% of patients reported that they are in direct contact with animals at their workplace or living place. Given that 90% of rural residents affected by brucellosis contacting to animals, this exposure can be seen as a key risk factor. Additionally, our findings reveal that about 65% of patients had a history of consuming raw dairy products. Previous studies reported raw dairy consumption as the most common risk factor for brucellosis, which differs from our results[18, 48, 49] In rural areas, there is a common belief that unprocessed dairy products are more nutritious than pasteurized ones. This perception increases the

preference for consuming raw dairy products, such as milk, yogurt, and cream[11]. Educating programs, particularly among women who are more involved in family nutrition, could enhance awareness and reduce the consumption of raw dairy products. Our findings indicate peak incidence between March and June, with a subsequent decrease until reaching its lowest level in December. The infection rate then rises again, reaches its peak in June. Similar studies conducted in Isfahan Province [34], Bejestan and Gonabad in Razavi Khorasan Province [33], and Zanjan Province[50], also reported the highest incidence of brucellosis in June and July. This seasonal increase is likely associated with the breeding season. During this period, contact with fetal secretions during and after childbirth, combined with the increased production and consumption of unpasteurized milk and dairy products in summer, contributes to a higher incidence of brucellosis[11, 38, 48]. To mitigate this, educational programs focusing on protective measures against contact with livestock and their secretions, as well as reducing the consumption of raw dairy products during the summer months, could be effective in preventing brucellosis.

The ~~results of the~~ spatial clustering analysis of human brucellosis incidence across 19 ~~in~~districts in ~~nineteen districts across~~ Neyshabur from 2015 to 2022 ~~, Iran,~~ revealed ~~the existence of a~~ spatial cluster in ~~each~~ all years ~~(~~excluding 2016~~)~~. ~~from 2015 to 2022.~~ The distribution of these clusters~~ed~~ ~~districts~~ was ~~similar~~ consistent over the ~~throughout the study~~ years, with high incidence rates often observed in the southwestern part of the city. This region is characterized by poorer and more isolated villages, which often lack adequate health infrastructure and monitoring, particularly considering livestock exchange between neighboring villages.~~.~~ This shows insufficient attention to livestock immunization, inadequate quarantine actions in villages with high brucellosis incidence, and a lack of effective monitoring of public health in these areas. Additionally, clusters with low incidence have consistently been

observed in the northern and northwestern regions over the years. The lower incidence of brucellosis in these areas is likely due to the low population density in the villages. This region, situated between the cities of Quchan and Khoshab in Razavi Khorasan Province, is known for its small population compared to other villages. Between 2017 and 2019, as well as in 2021, the Belherat district, located near high-incidence areas, was characterized by a low incidence of the disease. A limitation of our study is the inability to determine the reasons for this spatial variation in the southeastern part of Neyshabur during these years.

The findings revealed the existence of clusters in both southern and northern districts. The spatial clusters seem to be contingent on the condition of neighboring districts, while no spatial clusters were detected in the central districts. This may indicate that central districts did not have a similar incidence of human brucellosis cases. In the northern region, especially in the northwest, low-low clusters are observable almost every year. It seems that human brucellosis is non-endemic and does not pose a significant threat to the human population in these districts. The high incidence of human brucellosis cases was observed in southern regions, particularly in southwest districts. A High-High cluster

The results of Moran's spatial Autocorrelation (Global Moran's I) indicate that over the eight years studied, the distribution of malaria in Neyshabur's villages was random, with no consistent pattern of high or low incidence. This suggests that there is a need for more comprehensive and precise studies to better predict brucellosis incidence in Neyshabur City, especially given the rising trend of the disease. Enhanced and detailed research is necessary to address the increasing rates of brucellosis in this city.

was identified in 2017 and 2019. The analysis indicates that these districts harbor the potential for an outbreak of human brucellosis, posing a risk of significant and

irreversible health and economic repercussions for the local population. The results of the Spatial Autocorrelation analysis (Global Moran's I) of human brucellosis in Neyshabur from 2015 to 2022 suggest that the distribution of human brucellosis cases exhibits randomness and does not demonstrate a discernible spatial pattern. In reality, the incidence trend of human brucellosis does not adhere to a discernible pattern, thereby presenting challenges in forecasting future conditions.

Conclusions

The findings of this study can inform the planning of educational initiatives, particularly those targeting mothers, as well as strategies for animal vaccination. These efforts should be emphasized during the months leading up to the peak human brucellosis season and in regions with high incidence rates. Future research should explore the extent of livestock vaccination coverage and its correlation with the incidence of human brucellosis.

For the implementation of well prevention strategies Information about trends of incidence can be critical to management of resources in specific space and time. The results of our spatiotemporal cluster analysis demonstrate the presence of a distinct seasonality in human brucellosis. The period of primary prevalence extended from March to July, from 2015 to 2022 in Neyshabur, Iran. Given the heightened risk of contracting the disease through contact with blood, placenta, fetuses, and uterine secretions, it seems that these months present higher risk factors for human infection. Considering that the primary and most effective method to prevent Human

Brucellosis is to interrupt the transmission cycle from infected livestock to humans, the rate is much lower than anticipated. In addition, a majority of the patients have familial ties to animal husbandry as a traditional occupation in rural areas. Consequently, it is anticipated that in the forthcoming years, greater emphasis will be placed on livestock vaccination in rural areas with high infection rates. The consumption of raw dairy products among patients suggests a deficiency in education and awareness concerning diseases such as human brucellosis, particularly among women, children, and the elderly, who have limited direct contact with infected livestock. Therefore, there is a necessity for educational initiatives aimed at preventing the contraction of human brucellosis, with a specific focus on mothers, who hold a pivotal role in the family's nutritional well-being. It seems that optimizing livestock vaccination can reduce the likelihood of contracting brucellosis as an occupational hazard for livestock farmers while instructing the target groups to refrain from using unpasteurized dairy products can alleviate the risk of brucellosis transmission within families.

Limitations & Strength

Due to inadequate accurate registration of the livestock numbers by districts, the lack of access to local animal vaccination data based on the geographical distribution of livestock husbandry, and the deficiency in measuring the annual rainfall at the district

level in Neyshabur, the present study didn't examine the effect of these factors on the annual incidence rate of human brucellosis. Despite these limitations, we indicated the spatial pattern of human brucellosis incidence in Neyshabur and increased our knowledge about the most common causes of the disease in the human population. This information can be used as a scientific resource for revising prevalence strategies to control or eradicate human brucellosis in Neyshabur.

Acknowledgments

We would like to extend our sincere gratitude to Dr. Benyamin Hosseini for his valuable comments and suggestions that allowed us to improve the quality of the study. Additionally, we are especially grateful for the help of Dr. Ensiyeh Mollanoroozy for her expert editing support, who spent hours to strengthen the overall quality of the manuscript beyond correcting grammatical and spelling mistakes.

Funding

This study was supported by the Student Research Committee (SRC) funds of Neyshabur University of Medical Sciences (NUMS). The funders had no role in study design, data collection and analysis, decision to publish, or preparation of the manuscript.

References

1. Nicoletti P. Brucellosis: past, present and future. Prilozi. 2010;31(1):21-32.
2. Gwida M, Al Dahouk S, Melzer F, Rösler U, Neubauer H, Tomaso H. Brucellosis - regionally emerging zoonotic disease? Croat Med J. 2010;51(4):289-95.
3. Mirambo MM, Mgode GF, Malima ZO, John M, Mngumi EB, Mhamphi GG, Mshana SE. Seropositivity of Brucella spp. and Leptospira spp. antibodies among abattoir workers and meat vendors in the city of Mwanza, Tanzania: A call for one health approach control strategies. PLoS Negl Trop Dis. 2018;12(6):e0006600.
4. Dadar M, Shahali Y, Whatmore AM. Human brucellosis caused by raw dairy products: A review on the occurrence, major risk factors and prevention. Int J Food Microbiol. 2019;292:39-47.
5. Esmaeili H. Brucellosis in Islamic republic of Iran. Journal of medical bacteriology. 2014;3(3-4):47-57.
6. Hasanjani Roushan MR, Ebrahimpour S. Human brucellosis: An overview. Caspian J Intern Med. 2015;6(1):46-7.
7. Mirnejad R, Jazi FM, Mostafaei S, Sedighi M. Epidemiology of brucellosis in Iran: A comprehensive systematic review and meta-analysis study. Microb Pathog. 2017;109:239-47.
8. Soleimani A, Alizadeh S, Farshad MS, Kusha A, Mohamdzadeh M, Haghiri L, et al. Descriptive epidemiology of human brucellosis in east Azerbaijan, 2001-2009. Medical Journal of Tabriz University of Medical Sciences. 2012;34(1):63-9.
9. Mohammadian M, Mohammadian-Hafshejani A. Epidemiological characteristics and incidence rate of brucellosis over a period of 14 years in the Tiran-Karvan Township, Isfahan, Iran. Journal of Isfahan Medical School. 2014;32(293):1103-9.
10. Mostafavi E, Asmand M. Trend of brucellosis in Iran from 1991 to 2008. Iranian Journal of Epidemiology. 2012;8(1).
11. Norouzinezhad F, Erfani H, Norouzinejad A, Ghaffari F, Kaveh F. Epidemiological Characteristics and Trend in the Incidence of Human Brucellosis in Iran from 2009 to 2017. J Res Health Sci. 2021;21(4):e00535.
12. Norouzinezhad F, Erfani H, Norouzinejad A, Kaveh F, Ghaffari F. Epidemiological indices and trend of incidence of human brucellosis in Khorasan-e-Razavi Province from 2009 to 2016. Journal of Military Medicine. 2019;21(4):362-71.
13. Havas KA, Ramishvili M, Navdarashvili A, Hill AE, Tsanava S, Imnadze P, Salman MD. Risk factors associated with human brucellosis in the country of Georgia: a case-control study. Epidemiol Infect. 2013;141(1):45-53.
14. Franc KA, Krecek RC, Häsler BN, Arenas-Gamboa AM. Brucellosis remains a neglected disease in the developing world: a call for interdisciplinary action. BMC Public Health. 2018;18(1):125.
15. Babaie E, Alesheikh AA, Tabasi M. Spatial prediction of human brucellosis (HB) using a GIS-based adaptive neuro-fuzzy inference system (ANFIS). Acta Trop. 2021;220:105951.
16. Bosilkovski M, Edwards MS, Calderwood S. Brucellosis: epidemiology, microbiology, clinical manifestations, and diagnosis. Uptodate, United States. 2019.
17. Khalid HM. Seroprevalence and Associated Risk Factors of Brucellosis Among Human Population in Duhok City, Iraq. Infect Drug Resist. 2023;16:2805-11.
18. Zeinali M, Doosti S, Amiri B, Gouya MM, Godwin GN. Trends in the Epidemiology of Brucellosis Cases in Iran during the Last Decade. Iran J Public Health. 2022;51(12):2791-8.
19. wikipedia. Counties_of_Iran [Available from: https://en.wikipedia.org/wiki/Counties_of_Iran.
20. Hashemi SH, Gachkar L, Keramat F, Mamani M, Hajilooi M, Janbakhsh A, et al. Comparison of doxycycline-streptomycin, doxycycline-rifampin, and ofloxacin-rifampin in the treatment of brucellosis: a randomized clinical trial. Int J Infect Dis. 2012;16(4):e247-51.
21. Sofian M, Aghakhani A, Velayati AA, Banifazl M, Eslamifar A, Ramezani A. Risk factors for human brucellosis in Iran: a case-control study. Int J Infect Dis. 2008;12(2):157-61.
22. Bat-Erdene D, Chuang YC, Chuang KY. Brucellosis knowledge and preventive practices among herders in Western Mongolia. Zoonoses Public Health. 2019;66(1):133-9.
23. Tao Z, Chen Q, Chen Y, Li Y, Mu D, Yang H, Yin W. Epidemiological Characteristics of Human Brucellosis - China, 2016-2019. China CDC Wkly. 2021;3(6):114-9.

24. Halimi L, Bagheri N, Hoseini B, Hashtarkhani S, Goshayeshi L, Kiani B. Spatial analysis of colorectal cancer incidence in Hamadan Province, Iran: a retrospective cross-sectional study. Applied Spatial Analysis and Policy. 2020;13:293-303.
25. Shirzadi MR, Mohammadi P, Moradi G, Goodarzi E, Khazaei S, Moayed L, Khazaei Z. The Incidence and Geographical Distribution of Brucellosis in Iran Using Geographic Information System and Prediction of its Incidence in 2021. J Prev Med Hyg. 2021;62(3):E635-e4.
26. Azizi Mayvan F, Hashtarkhani S, Latifi M, Darrudi R, Hoseini B. Geographic disparities of brucellosis distribution in Neyshabur between 2014 and 2016: a cross-sectional study based on geographical information systems. J Neyshabur Univ Med Sci. 2019;7(4):53-64.
27. Census of the Islamic Republic of Iran, 1395 (2016)". AMAR (in Persian). The Statistical Center of Iran 2016 [Available from: https://web.archive.org/web/20220402220537/https://www.amar.org.ir/.
28. contributors O. Nishapur County 2023 [Available from: https://geohack.toolforge.org/geohack.php?pagename=Nishapur_County&params=36_25_26_N_58_37_31_E_dim:60km_type:city(451780)_region:IR-09.
29. Anselin L. Local indicators of spatial association—LISA. Geographical analysis. 1995;27(2):93-115.
30. Amsalu ET, Akalu TY, Gelaye KA. Spatial distribution and determinants of acute respiratory infection among under-five children in Ethiopia: Ethiopian Demographic Health Survey 2016. PloS one. 2019;14(4):e0215572.
31. Azizi MF, Hashtarkhani S, Latifi M, Darrudi R, Hoseini B. Geographic disparities of brucellosis distribution in Neyshabur between 2014 and 2016: a cross-sectional study based on geographical information systems. 2020.
32. Bagheri H, Tapak L, Karami M, Amiri B, Cheraghi Z. Epidemiological Features of Human Brucellosis in Iran (2011-2018) and Prediction of Brucellosis with Data-Mining Models. J Res Health Sci. 2019;19(4):e00462.
33. Riabi HRA, Riabi HRA, Razmara H. Epidemiological Feature of the Human Brucellosis Prevalence in People in Southern Cities of Khorasan Razavi, Iran. Zahedan Journal of Research in Medical Sciences. 2017;19(4).
34. Salahshouri A, Ramezanpour J, Gheibipour H. Epidemiological Study of the Brucellosis in Iran, Isfahan, 2010–2015. Epidemiology and Health System Journal. 2021;8(3):95-9.
35. Kamal S, Hashemi S, Nasaji M, Moshiri E, Shahriyari R, Azizi A. Frequency of reported cases of brucellosis to province health center from public and private sectors in Semnan 2006-2007. 2009.
36. Pakzad R, Pakzad I, Safiri S, Shirzadi MR, Mohammadpour M, Behroozi A, et al. Spatiotemporal analysis of brucellosis incidence in Iran from 2011 to 2014 using GIS. Int J Infect Dis. 2018;67:129-36.
37. Sun ZX, Wang Y, Li YJ, Yu SH, Wu W, Huang DS, Guan P. Socioeconomic, meteorological factors and spatiotemporal distribution of human brucellosis in China between 2004 and 2019-A study based on spatial panel model. PLoS Negl Trop Dis. 2023;17(11):e0011765.
38. Moosazadeh M, Abedi G, Kheradmand M, Safiri S, Nikaeen R. Seasonal pattern of brucellosis in Iran: A systematic review and meta-analysis. 2016.
39. Gebreyes WA, Dupouy-Camet J, Newport MJ, Oliveira CJ, Schlesinger LS, Saif YM, et al. The global one health paradigm: challenges and opportunities for tackling infectious diseases at the human, animal, and environment interface in low-resource settings. PLoS Negl Trop Dis. 2014;8(11):e3257.
40. Godfroid J. Brucellosis in livestock and wildlife: zoonotic diseases without pandemic potential in need of innovative one health approaches. Arch Public Health. 2017;75:34.
41. Hayoun MA ME, Shorman M. Brucellosis 2023 [Available from: Hayoun MA, Muco E, Shorman M. Brucellosis. [Updated 2023 Apr 29]. In: StatPearls [Internet]. Treasure Island (FL): StatPearls Publishing; 2023 Jan-. Available from: https://www.ncbi.nlm.nih.gov/books/NBK441831/.
42. Abdelkader A, Reda BA, Karima G. Prevalence of human brucellosis in the southern Zone of Sidi-Bel-Abbes, Algeria. Archives of Clinical and Medical Case Reports. 2018;2(2):56-64.
43. Mufinda FC, Boinas F, Nunes C. Prevalence and factors associated with human brucellosis in livestock professionals. Rev Saude Publica. 2017;51:57.
44. Alqaseer K, Al-Khafajy AAM, Almkhadhree EAK. Serological and Molecular Detection of Human Brucellosis in Rural Areas in Wasit Province, Iraq. Arch Razi Inst. 2023;78(1):369-78.

45. Hassan L, Ali S, Syed MA, Shah AA, Abbasi SA, Tabassum S, et al. Risk Factors for Acute Brucellosis in Patients on the Day of Admission at Selected Hospitals of Abbottabad, Pakistan. Front Public Health. 2021;9:669278.
46. Elbeltagy KE. An epidemiological profile of brucellosis in Tabuk Province, Saudi Arabia. East Mediterr Health J. 2001;7(4-5):791-8.
47. Liu B, Liu G, Ma X, Wang F, Zhang R, Zhou P, et al. Epidemiology, clinical manifestations, and laboratory findings of 1,590 human brucellosis cases in Ningxia, China. Front Microbiol. 2023;14:1259479.
48. Pordanjani SR, Atamaleki A, Amiri M, Khazaei Z, Fallahzadeh H, Alayi R, et al. Study on epidemiological status, spatial and temporal distribution of human brucellosis in kohgiluyeh and Boyer-Ahmad Province during 2011–2017. Advances in Human Biology. 2020;10(1):22-8.
49. Dehghan A, Sadeghian M, Jafarnejad A. Epidemiologic survey of Brucellosis in Fasa during 2009-2017. medical journal of mashhad university of medical sciences. 2019;62(2):1449-55.
50. Rostami Z, Faghihzadeh S, Tagilou B, Khosravi Y. Determining the Incidence Rate and Risk Factors of Brucellosis in Zanjan Province (Iran) from 2012 to 2017: A Spatiotemporal Analysis. 2020.

- You can change the title to news and mention the type of study in the title

+ Dear Reviewer, the type of study is clearly stated in the title of the article

- You can write Neyshabur, a city in the east of Iran

+ Dear Reviewer, due to the lengthy title of the article, this issue has also been addressed in the abstract and methods sections

- The working method of the study is written in the abstract in a very limited way. The exact method of implementation should be written

+Dear Reviewer, the summary of the methods section of the article includes all four main components: Study Area, Data Sources and Patients, Ethics Approval and Consent to Participate, and Statistical Analysis, which are also mentioned in the abstract

- In the conclusion section for this study, it should be written and applied

+ Dear Reviewer, the operational application of the results has been addressed in the conclusion

- Match keywords with mesh

+ Dear Reviewer, keywords were matched with MeSH and modified

- In the introduction, it should be explained how most brucellosis is transmitted and how it is in Iran

+Dear Reviewer, the introduction of the article discusses the mode of transmission of the pathogenic agent of Malt fever, as well as the common causes of transmission among the Iranian population.

- Where are the most involved areas in Iran?

+ Dear Reviewer, human brucellosis in Iran is regarded as a widespread disease throughout the country. According to previous studies referenced in the article, the highest number of patients is reported in Razavi Khorasan Province, located in the northeast of Iran. Additionally, the incidence rate in the northwestern regions is higher due to the smaller population residing in these provinces.

- Write the occurrence and prevalence by mentioning the figures

+ Dear Reviewer, the introduction references similar studies conducted in other regions of the province and throughout Iran, highlighting the incidence rates.

- There was no explanation given in the introduction about what was the reason to do this study in Neishabur

+ Dear Reviewer, the introduction notes that Neyshabur is the second largest city in the province. Due to the significant number of villages and the markedly higher prevalence of human brucellosis in rural areas, previous studies conducted in Neyshabur consistently reported high incidence rates. To assess the impact of these prior studies and the changes in planning for disease control and containment, this study was conducted over an extended period. Additionally, due to the general lifestyle in Iran being similar to that of a village, the results of this study can be applicable to other regions, particularly the cities and villages of Razavi Khorasan Province

- Most of the information and statistics are for 2017, you can update it until 2023

+ Dear Reviewer, new studies have been reviewed and referenced in the introduction

- Write down other studies that exist in this field especially in the east of Iran e the occurrence and prevalence by mentioning the figures

+ Dear Reviewer, the results of studies conducted in Razavi Khorasan Province and neighboring provinces are discussed and compared in the discussion section.

- Write the criteria for entering the study

+ Dear Reviewer, the criteria for identifying patients as cases of human malaria are outlined in the methods section.

- Rewrite the study implementation method section in the work method, it is written very vaguely and scattered

+ Dear Reviewer, the method has been rewritten, and an effort has been made to present it more clearly.

- In the results section, you have only brought a table, while you can bring some findings in the form of a table

+ Dear Reviewer, the results are presented in two separate tables

- The discussion of the study is also written very briefly

+ Dear Reviewer, the discussion section of the article has been rewritten in accordance with the reviewers' feedback. New studies have been reviewed and compared, and efforts have been made to provide a more comprehensive interpretation of the results obtained

- In the discussion after your findings, the author's own analysis should be written in this context

+ Dear Reviewer, the authors' personal interpretation of the results obtained has been included

- Bring studies both similar and contradictory to these results

+ Dear Reviewer, studies that are similar to and in contrast with the findings of the present study have been reviewed and presented, focusing on incidence rates based on gender, place of residence, age, and different times of the year

- In the discussion part, the results of all the cities in the field of Brucellosis, especially the province in question and the neighboring cities, should be used

+ Dear Reviewer, the results of similar studies conducted in the cities of Quchan, Khoshab, and Behbahan in Razavi Khorasan Province have been mentioned. Studies related to the distant past were not discussed, as they fall outside the time frame examined in this study

- The conclusion is too long

+ Dear Reviewer, I have attempted to summarize the abstract and rewrite it by highlighting the practical applications of the study

- Focus on the result of this study and its applications

+ Dear Reviewer, the practical results of the present study have been highlighted in relation to organizational planning

- Mention the suggestions in this context

+ Dear Reviewer, a suggestion has been made to conduct a future study to build upon the present research.

- More for the results of your own study, you should continue drawing conclusions

+ Dear Reviewer has made efforts to provide a more comprehensive conclusion and interpretation of the results compared to the previous version

In the introduction, researchers should justify the necessity of spatio-temporal analysis of brucellosis in Neyshabur. Neyshabur is a small city in the east of Iran. The main centers of brucellosis are concentrated in the western provinces of Iran. What are the spatial units in Neishabur city?

+ Dear Reviewer, Neyshabur is the second largest city in Razavi Khorasan Province, encompassing over 350 villages within its geographical area. The results indicate that more than 90% of the infected individuals reside in rural areas, where the population largely maintains a traditional lifestyle. The human brucellosis infection rate over the eight-year study period shows a significantly higher prevalence in Neyshabur compared to other cities in the province. Identifying areas with high or low infection rates during this period can serve as a roadmap for planners to allocate resources more effectively to prevent human brucellosis. An upward trend in annual infections has been observed in most years, indicating a lack of success in current implementation programs in this city. This study serves as a foundation for future research aimed at successfully controlling and containing human brucellosis in Neyshabur

Due to the fact that the result was incomplete and I did not have access to the figures, I cannot fully review the article.

+ Dear Reviewer, in accordance with the journal's guidelines for submitting images, the images related to the results were initially sent in a separate file in the supporting information section. In the current version, the images have been placed in their relevant sections alongside the results
