# Supplementary material for: Spatio-temporal analysis of human brucellosis distribution in Neyshabur, Iran from 2015 to 2022, a cross-sectional study
Source: PLoS One. 2025 Feb 18;20(2):e0318503. doi: 10.1371/journal.pone.0318503 (PMC11835317; doi:10.1371/journal.pone.0318503)
Supplement: Attachment — Submitted filename: Comments.docx [file pone.0318503.s001.docx]

1. In the introduction, researchers should justify the necessity of spatio-temporal analysis of brucellosis in Neyshabur. Neyshabur is a small city in the east of Iran. The main centers of brucellosis are concentrated in the western provinces of Iran. What are the spatial units in Neishabur city?
2. Due to the fact that the result was incomplete and I did not have access to the figures, I cannot fully review the article.
