# Supplementary material for: Spatio-temporal analysis of human brucellosis distribution in Neyshabur, Iran from 2015 to 2022, a cross-sectional study
Source: PLoS One. 2025 Feb 18;20(2):e0318503. doi: 10.1371/journal.pone.0318503 (PMC11835317; doi:10.1371/journal.pone.0318503)
Supplement: Attachment — Submitted filename: Response to Reviewers.docx [file pone.0318503.s003.docx]

- You can change the title to news and mention the type of study in the title

+ Dear Reviewer, the type of study is clearly stated in the title of the article

- You can write Neyshabur, a city in the east of Iran

+ Dear Reviewer, due to the lengthy title of the article, this issue has also been addressed in the abstract and methods sections

- The working method of the study is written in the abstract in a very limited way. The exact method of implementation should be written

+ Dear Reviewer, the summary of the methods section of the article includes all four main components: Study Area, Data Sources and Patients, Ethics Approval and Consent to Participate, and Statistical Analysis, which are also mentioned in the abstract

- In the conclusion section for this study, it should be written and applied

+ Dear Reviewer, the operational application of the results has been addressed in the conclusion

- Match keywords with mesh

+ Dear Reviewer, keywords were matched with MeSH and modified

- In the introduction, it should be explained how most brucellosis is transmitted and how it is in Iran

+ Dear Reviewer, the introduction of the article discusses the mode of transmission of the pathogenic agent of Malt fever, as well as the common causes of transmission among the Iranian population.

- Where are the most involved areas in Iran?

+ Dear Reviewer, human brucellosis in Iran is regarded as a widespread disease throughout the country. According to previous studies referenced in the article, the highest number of patients is reported in Razavi Khorasan Province, located in the northeast of Iran. Additionally, the incidence rate in the northwestern regions is higher due to the smaller population residing in these provinces.

- Write the occurrence and prevalence by mentioning the figures

+ Dear Reviewer, the introduction references similar studies conducted in other regions of the province and throughout Iran, highlighting the incidence rates.

- There was no explanation given in the introduction about what was the reason to do this study in Neishabur

+ Dear Reviewer, the introduction notes that Neyshabur is the second largest city in the province. Due to the significant number of villages and the markedly higher prevalence of human brucellosis in rural areas, previous studies conducted in Neyshabur consistently reported high incidence rates. To assess the impact of these prior studies and the changes in planning for disease control and containment, this study was conducted over an extended period. Additionally, due to the general lifestyle in Iran being similar to that of a village, the results of this study can be applicable to other regions, particularly the cities and villages of Razavi Khorasan Province

- Most of the information and statistics are for 2017, you can update it until 2023

+ Dear Reviewer, new studies have been reviewed and referenced in the introduction

- Write down other studies that exist in this field especially in the east of Iran e the occurrence and prevalence by mentioning the figures

+ Dear Reviewer, the results of studies conducted in Razavi Khorasan Province and neighboring provinces are discussed and compared in the discussion section.

- Write the criteria for entering the study

+ Dear Reviewer, the criteria for identifying patients as cases of human malaria are outlined in the methods section.

- Rewrite the study implementation method section in the work method, it is written very vaguely and scattered

+ Dear Reviewer, the method has been rewritten, and an effort has been made to present it more clearly.

- In the results section, you have only brought a table, while you can bring some findings in the form of a table

+ Dear Reviewer, the results are presented in two separate tables

- The discussion of the study is also written very briefly

+ Dear Reviewer, the discussion section of the article has been rewritten in accordance with the reviewers' feedback. New studies have been reviewed and compared, and efforts have been made to provide a more comprehensive interpretation of the results obtained

- In the discussion after your findings, the author's own analysis should be written in this context

+ Dear Reviewer, the authors' personal interpretation of the results obtained has been included

- Bring studies both similar and contradictory to these results

+ Dear Reviewer, studies that are similar to and in contrast with the findings of the present study have been reviewed and presented, focusing on incidence rates based on gender, place of residence, age, and different times of the year

- In the discussion part, the results of all the cities in the field of Brucellosis, especially the province in question and the neighboring cities, should be used

+ Dear Reviewer, the results of similar studies conducted in the cities of Quchan, Khoshab, and Behbahan in Razavi Khorasan Province have been mentioned. Studies related to the distant past were not discussed, as they fall outside the time frame examined in this study

- The conclusion is too long

+ Dear Reviewer, I have attempted to summarize the abstract and rewrite it by highlighting the practical applications of the study

- Focus on the result of this study and its applications

+ Dear Reviewer, the practical results of the present study have been highlighted in relation to organizational planning

- Mention the suggestions in this context

+ Dear Reviewer, a suggestion has been made to conduct a future study to build upon the present research.

- More for the results of your own study, you should continue drawing conclusions

+ Dear Reviewer has made efforts to provide a more comprehensive conclusion and interpretation of the results compared to the previous version

In the introduction, researchers should justify the necessity of spatio-temporal analysis of brucellosis in Neyshabur. Neyshabur is a small city in the east of Iran. The main centers of brucellosis are concentrated in the western provinces of Iran. What are the spatial units in Neishabur city?

+ Dear Reviewer, Neyshabur is the second largest city in Razavi Khorasan Province, encompassing over 350 villages within its geographical area. The results indicate that more than 90% of the infected individuals reside in rural areas, where the population largely maintains a traditional lifestyle. The human brucellosis infection rate over the eight-year study period shows a significantly higher prevalence in Neyshabur compared to other cities in the province. Identifying areas with high or low infection rates during this period can serve as a roadmap for planners to allocate resources more effectively to prevent human brucellosis. An upward trend in annual infections has been observed in most years, indicating a lack of success in current implementation programs in this city. This study serves as a foundation for future research aimed at successfully controlling and containing human brucellosis in Neyshabur

Due to the fact that the result was incomplete and I did not have access to the figures, I cannot fully review the article.

+ Dear Reviewer, in accordance with the journal's guidelines for submitting images, the images related to the results were initially sent in a separate file in the supporting information section. In the current version, the images have been placed in their relevant sections alongside the results
