# Supplementary material for: Spatio-temporal analysis of human brucellosis distribution in Neyshabur, Iran from 2015 to 2022, a cross-sectional study
Source: PLoS One. 2025 Feb 18;20(2):e0318503. doi: 10.1371/journal.pone.0318503 (PMC11835317; doi:10.1371/journal.pone.0318503)
Supplement: Attachment — Submitted filename: Response_to_Reviewers_.docx [file pone.0318503.s005.docx]

-You should not write the code of ethics in the abstract part of the work procedure; you should put it in the appropriate place at the end of the conclusion

+ Dear Reviewer, Thank you for your valuable feedback. I have removed the code of ethics from the methods section in the abstract and placed it in the acknowledgments section of the study as suggested

- In the results section of the abstract, use the statistics of the results section

+ Thank you for your insightful comments. In response to your suggestion, I have incorporated more statistical data from the results section into the abstract.

- Match the key blocks with the mesh

+ Thank you for your feedback. I have matched all key terms with the appropriate MeSH (Medical Subject Headings) terms in PubMed to ensure accurate indexing and categorization

- Complete the texts in the introduction section and use more relevant studies in this field

+ Thank you for your constructive feedback. I have expanded the introduction section by incorporating additional relevant studies in this field to enhance its completeness and depth.

- In the introduction section, write the importance and reasons for conducting the study

+ Thank you for your valuable comments. In response, I have emphasized the importance and rationale for conducting the study in Neyshabur within the introduction section. This includes addressing the necessity of the study in relation to the specific time, location, and type of research, as well as the overall purpose of conducting this study at this time.

- The text of the article needs English editing and writing, especially in the results and discussion and working methods

+ Thank you for your feedback. I have had the article revised by an English editor to improve the clarity and quality of the writing, particularly in the results, discussion, and methods sections.

-More internal and external studies should be used in the discussion

+ Thank you for your insightful comments. In response, I have enhanced the discussion section by incorporating comparisons with additional recent internal and external studies to provide a more comprehensive analysis of the results.

- Use both similar and contradictory results

+ Thank you for your valuable feedback. In response, I have included a discussion of both similar and contradictory studies in the discussion section to provide a more balanced perspective on the results.

- Write the limitations of the study

+ done.

- Write suggestions and application

+ Thank you for your constructive comments. In response, I have added a suggestions and application section following the limitations and strengths section to provide practical recommendations based on the study findings.

Dear reviewer,

Thank you for your valuable comments and suggestions on my manuscript. Your insights have greatly improved the clarity and quality of the work.

I appreciate your time and effort in reviewing my article.

Best regards,
