# Supplementary material for: Spatio-temporal analysis of human brucellosis distribution in Neyshabur, Iran from 2015 to 2022, a cross-sectional study
Source: PLoS One. 2025 Feb 18;20(2):e0318503. doi: 10.1371/journal.pone.0318503 (PMC11835317; doi:10.1371/journal.pone.0318503)
Supplement: Attachment — Submitted filename: Response_to_Reviewers_auresp_3.docx [file pone.0318503.s006.docx]

Reviewer #3: Have the authors made all data underlying the findings in their manuscript fully available? No
The authors have declared some level of restrictions to data availability.

4. Have the authors made all data underlying the findings in their manuscript fully available?

The [PLOS Data policy](http://track.editorialmanager.com/CL0/http:%2F%2Fwww.plosone.org%2Fstatic%2Fpolicies.action%23sharing/1/010f01942b26d4f3-b3820cef-b53a-442e-aeb5-20c553f7ff3c-000000/npFWfRXAXxJB3Qh8126DphLDuUtwMgMkMrP-ibD2-Rs=191) requires authors to make all data underlying the findings described in their manuscript fully available without restriction, with rare exception (please refer to the Data Availability Statement in the manuscript PDF file). The data should be provided as part of the manuscript or its supporting information, or deposited to a public repository. For example, in addition to summary statistics, the data points behind means, medians and variance measures should be available. If there are restrictions on publicly sharing data—e.g. participant privacy or use of data from a third party—those must be specified.

Reviewer #1: Yes

Reviewer #3: No

+ Thank you for your inquiry regarding data sharing. We appreciate the importance of transparency and accessibility in research.

The data used in this study was obtained from the Neyshabur Health Center as offline documents and Excel files. As the authors do not own this data, we are unable to provide a direct link or public access. Additionally, the Neyshabur Health Center is a local service-oriented institution that lacks the infrastructure for handling international correspondence, particularly in languages other than Persian. There is currently no established protocol for responding to data requests from international researchers, which limits our ability to facilitate direct contact for data access.

Moreover, the data includes sensitive information, such as geographical coordinates of patients, imposing further restrictions on sharing due to privacy and ethical considerations.

Despite these constraints, we are committed to sharing the data with individual researchers upon request, provided that such requests comply with the ethical guidelines and constraints under which the data was collected. The data will be stored securely in a personal and online encrypted cloud repository to ensure long-term availability.

While we cannot make our study’s data publicly available at the time of publication, we assure you that all authors are committed to making the data underlying the findings described in this study fully available without restriction to those who request it, in compliance with the PLOS Data Availability policy. For datasets involving personally identifiable information or other sensitive data, data sharing will be contingent on the data being handled appropriately by the requester and in accordance with all applicable local requirements.

Thank you for your understanding.
